# Hyperphosphorylated tau causes reduced hippocampal CA1 excitability by relocating the axon initial segment

**DOI:** 10.1007/s00401-017-1674-1

**Published:** 2017-01-16

**Authors:** Robert John Hatch, Yan Wei, Di Xia, Jürgen Götz

**Affiliations:** 1 Clem Jones Centre for Ageing Dementia Research, Queensland Brain Institute, The University of Queensland, St Lucia Campus, Brisbane, QLD 4072 Australia; 2 State Key Laboratory of Brain and Cognitive Science, Institute of Biophysics, Chinese Academy of Sciences, Beijing, 100101 China

**Keywords:** Axon initial segment, Action potential, Tau, Hippocampus, CA1, Neurodegeneration

## Abstract

Hyperphosphorylated tau has a critical role in tauopathies such as Alzheimer’s disease and frontotemporal dementia, impairing neuronal function and eventually leading to neurodegeneration. A critical role for tau is supported by studies in transgenic mouse models that express the P301L tau mutation found in cases of familial frontotemporal dementia, with the accumulation of hyperphosphorylated tau in the hippocampus causing reductions in hippocampal long-term potentiation and impairments in spatial learning and memory. However, what has remained unexplored is the role of hyperphosphorylated tau in reducing neuronal excitability. Here, we show in two complementary P301L tau transgenic mouse models that hyperphosphorylated tau induces a more depolarized threshold for action potential initiation and reduces firing in hippocampal CA1 neurons, which was rescued by the suppression of transgenic tau. Furthermore, using mutagenesis and primary hippocampal neuronal cultures, we reveal that this reduction in neuronal excitability results from the relocation of the axon initial segment (AIS) down the axon in a tau phosphorylation-dependent manner. We also demonstrate that this effect is microtubule-dependent. In addition, pharmacological stabilization was found to prevent both the structural and functional deficits caused by tau hyperphosphorylation. Finally, we demonstrate that the AIS of neurons from tau transgenic mice is further down the axon, which correlates with a reduction in excitability. We therefore propose that a reduction in hippocampal excitability due to a tau-mediated distal relocalization of the AIS contributes to the hippocampal dysfunction observed in tauopathies.

**Electronic supplementary material:**

The online version of this article (doi:10.1007/s00401-017-1674-1) contains supplementary material, which is available to authorized users.

## Introduction

The intracellular accumulation of the microtubule-associated protein tau in a hyperphosphorylated form characterizes many neurodegenerative diseases, including Alzheimer’s disease (AD) and major forms of frontotemporal lobar degeneration (FTLD-Tau) [23]. Although tau accumulation is central to these diseases, the mechanism whereby pathological tau impairs neuronal function is incompletely understood. To address this issue, a range of transgenic animal models have been generated that express different mutant forms of tau found in familial cases of FTLD-Tau [12, 15, 22]. Expression of these forms of tau recapitulates major aspects of the human pathology, including hyperphosphorylation, neurofibrillary tangle (NFT) formation, neurodegeneration and impaired hippocampal-dependent spatial memory, as demonstrated by the rTg4510 mouse strain that expresses the human P301L mutation of FTLD-Tau [21, 31, 36, 40, 45]. Furthermore, using the rTg4510 mouse model, it has been shown that the early stage of tau aggregation and hyperphosphorylation which precedes NFT formation strongly correlates with neuronal dysfunction [2–4, 31, 43, 45]. To identify how this occurs, numerous studies have focussed on the involvement of tau impaired synaptic activity [5, 21, 24, 29]. However, its impact on neuronal excitability has received less attention, although it has been reported that the removal of tau reduces network hyperexcitability [7, 20, 42], suggesting an important role in the modulation of neuronal excitability. Here, we used patch-clamp electrophysiology of hippocampal CA1 neurons in two tau pathology mouse models, the rTg4510 strain and a second model, pR5, that also expresses P301L mutant Tau, albeit at much lower levels [14]. Electrophysiological recordings in brain slices were complemented by an analysis of primary hippocampal neurons that were transfected with different forms of tau, both mimicking and abrogating hyperphosphorylation, to determine if hyperphosphorylated tau reduces neuronal excitability. CA1 hippocampal rather than cortical neurons were investigated as previous reports have revealed a role for hyperphosphorylated tau in impairing hippocampus-dependent memory functions in animal studies [21, 37, 40]. This allowed us to identify a critical role for hyperphosphorylated tau in impairing hippocampal neuronal excitability by distally relocating the axon initial segment (AIS) in a microtubule-dependent manner that preceded neurodegeneration.

## Methods

### Ethics statement and mouse strains

All experimental procedures were conducted under the guidelines of the Australian Code of Practice for the Care and Use of Animals for Scientific Purposes and were approved by the University of Queensland Animal Ethics Committee (QBI/412/14/NHMRC; QBI/027/12/NHMRC). Experiments and data analysis were performed blind to the experimental group. Mice were maintained on a 12-h light/dark cycle and housed in a PC2 facility with ad libitum access to food and water. In addition to wild-type mice, two P301L mutant human tau transgenic mouse strains were used: the inducible rTg4510 strain that is characterized by a 13-fold overexpression of human tau compared to endogenous tau [45], and the pR5 strain that expresses human tau at approximately 70% of the levels of endogenous mouse tau [13]. Male and female mice were used at an equal ratio and were randomly allocated to experimental groups. To suppress transgenic tau expression, rTg4510 mice were fed ad libitum a chow diet containing doxycycline (625 mg/kg) for 4 weeks, as previously described [45].

### Brain slice preparation

Acute brain slices were prepared similar to previous reports [18, 35]. rTg4510 [45], pR5 [14] and wild-type control mice were anesthetized with 2% isoflurane (Attane) and transcardially perfused with cold cutting solution comprising (in mM) 125 choline-Cl, 2.5 KCl, 0.4 CaCl_2_, 6 MgCl_2_, 1.25 NaH_2_PO_4_, 26 NaHCO_3_ and 20 d-glucose saturated with 95% O_2_ and 5% CO_2_. The mice were then decapitated and the brain quickly removed. 300 µm coronal hippocampal brain slices were cut on a vibratome (VT1000S, Leica). Slices were rested for 30 min at 35 °C and then at room temperature (RT) for at least 30 min prior to electrophysiological recordings.

### Whole-cell patch-clamp electrophysiology

**Fig. 1:**
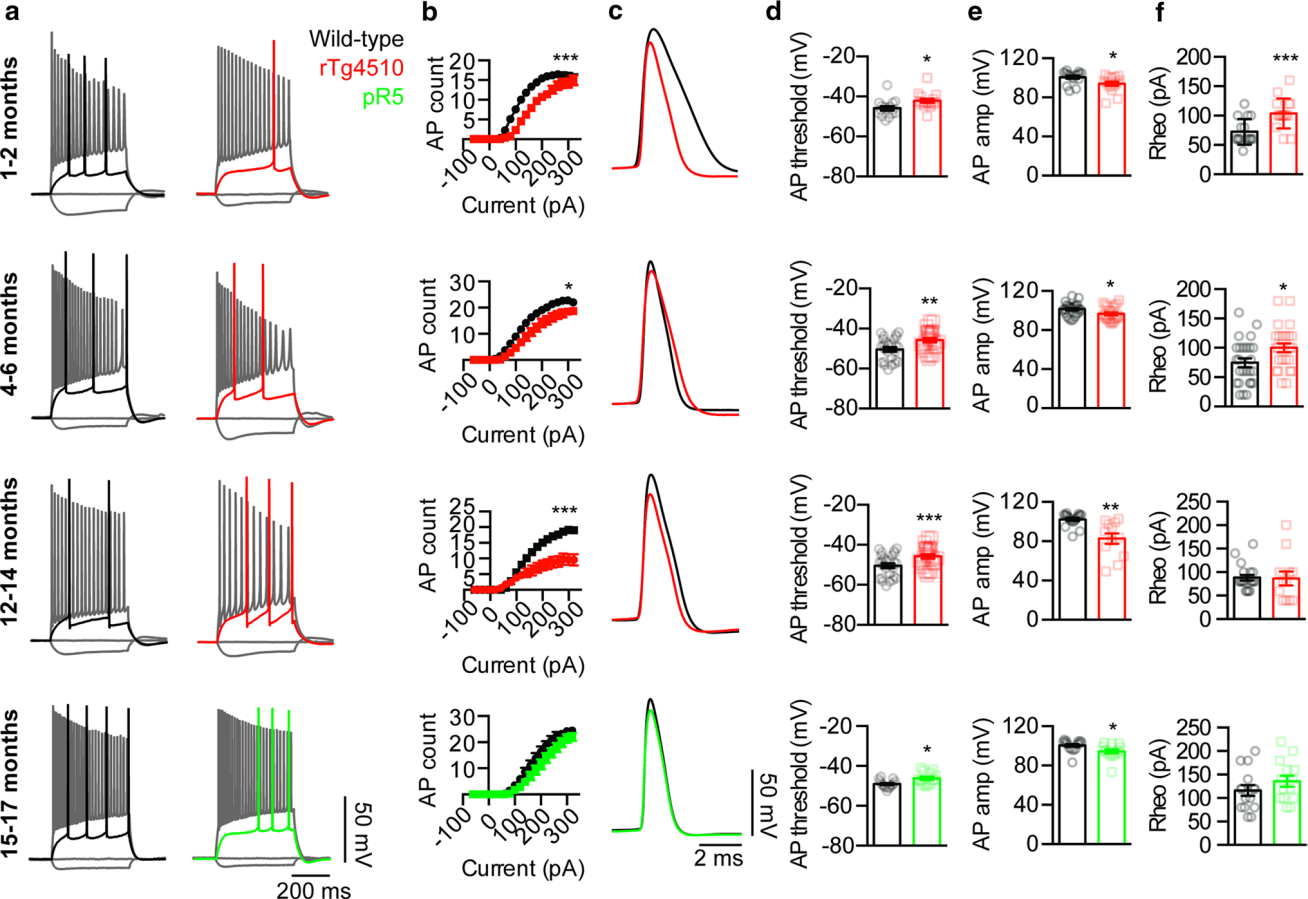
Neuronal excitability is reduced in rTg4510 and pR5 CA1 pyramidal neurons. **a** Representative traces of APs fired by rTg4510 (*red*), pR5 (*green*) and wild-type (*black*) neurons following injection of a −60, 0, rheobase and 320 pA current step. **b** Input–output relationships from rT4510 mice aged 1–2 months (*top row*), 4–6 months (*second row*), and 12–14 months (*third row*) as well as pR5 mice aged 15–17 months (*fourth row*) and wild-type age-matched control neurons (*top row* rTg4510 *n* = 16 neurons *n* = 5 mice, wild-type *n* = 16 neurons *n* = 3 mice, *p* = 0.0004; *second row* rTg4510 *n* = 24 neurons *n* = 4 mice, wild-type *n* = 26 neurons *n* = 4 mice, *p* = 0.0124; *third row* rTg4510 *n* = 12 neurons *n* = 5 mice, wild-type *n* = 20 neurons *n* = 4 mice, *p* = 0.0003; *fourth row* pR5 *n* = 9 neurons *n* = 3 mice, wild-type *n* = 11 neurons *n* = 3 mice, *p* = 0.15). **c** Representative traces of the first APs fired in rT4510 and wild-type control neurons. Pooled data demonstrating the AP firing of neurons from transgenic tau neurons compared to wild-type controls, including **d** AP threshold (*top row p* = 0.0148; *second row p* = 0.0044; *third row p* = 0.0002; *fourth row p* = 0.0313), (**e**) AP amplitude (*top row p* = 0.0147; *second row p* = 0.0188; *third row p* = 0.0002; *fourth row p* = 0.0499), and **f** rheobase (*top row p* = 0.0008; *second row p* = 0.0124; *third row p* = 0.90; *fourth row p* = 0.23). *p < 0.05, **p < 0.01, ***p < 0.001. Data presented as mean ± SEM and individual data points. Statistical comparisons were made using an unpaired two-tailed Student’s *t* test

Slices were transferred to a submerged recording chamber on an upright microscope (Slicescope Pro 1000; Scientifica) and perfused with an oxygenated recording artificial cerebrospinal fluid solution comprising (in mM) 125 NaCl, 2.5 KCl, 2 CaCl_2_, 2 MgCl_2_, 1.25 NaH_2_PO_4_, 26 NaHCO_3_, 10 d-glucose at 32 °C. CA1 neurons were identified visually, using infrared-oblique illumination microscopy with a 40× water-immersion objective (Olympus) and a CCD camera (Jenoptik, Optical Systems GmbH) as well as by their action potential (AP) firing characteristics: pyramidal neurons were found in the *stratum pyramidale* of the CA1 region and displayed AP firing that was accommodating at high current injections and had a wide AP (see Fig. 1) [34, 48], whereas fast-spiking inhibitory interneurons were found in the *stratum oriens* of the CA1 region and fired non-accommodating AP trains at high stimulation currents with a narrow AP width (see Supplementary Figure 3) [11, 27, 49]. Transfected hippocampal primary neuron cultures were perfused with a HEPES-buffered external solution composed of (in mM) 140 NaCl, 5 KCl, 2 CaCl_2_, 1 MgCl_2_, 10 HEPES, and 10 d-glucose (pH 7.4) at 25 °C and were identified based on enhanced green fluorescent protein (EGFP) expression. Whole-cell patch-clamp recordings were performed using a micro-manipulator (Scientifica) and an Axon MultiClamp 700B patch-clamp amplifier (Molecular Devices). Data were acquired using pClamp software (v10; MDS) with a sampling rate of 50 kHz after Bessel filtering at 10 kHz (Digidata 1440a; Axon). Patch pipettes (4–7 MΩ; GC150F-10; Harvard Instruments) were pulled using a micropipette puller (PC-10; Narishige) and then filled with an internal solution containing (in mM) 125 K-gluconate, 5 KCl, 2 MgCl_2_·6H_2_0, 10 HEPES, 4 ATP-Mg, 0.3 GTP-Na, 10 phosphocreatine, 10 EGTA and 0.2% biocytin (pH 7.24 and 291 mOsm). Neuronal capacitance and input resistance were determined in voltage-clamp mode with the cells being held at −70 mV for neurons from brain slices and −60 mV for primary cultures. Neuronal excitability was determined in current-clamp mode, with a holding current injected to maintain the membrane potential at approximately −70 mV for neurons in brain slices and −60 mV for primary cultures. Current steps were then injected to generate AP firing.

### Electrophysiology data analysis

Data were analyzed using the Axograph X software. Individual APs were identified using a 50 mV/ms threshold for neurons from brain slices and 10 mV/ms for primary cultured neurons. Raw traces were normalized by baseline subtraction. Input–output relationships were calculated by determining the number of APs that were generated for each injected current step. The integrated AP firing, which was used to compare AP firing between groups, was calculated from the area underneath individual input–output relationships for all current steps. All analysis of AP morphology was calculated from the first AP fired. The threshold for AP firing was determined, prior to baseline subtraction, at the membrane potential at which the threshold was reached. The rheobase current was determined as the first current step from which an AP was generated. The AP amplitude and after hyperpolarization (AHP) were calculated relative to threshold. The AP rise-time was calculated as the time between 10 and 90% of the maximal AP amplitude. The AP half-width was determined at 50% of the maximal AP amplitude. The sag potential was calculated by subtracting the membrane voltage at the beginning of a −60 pA, 400 ms hyperpolarizing current step from the steady state [35].

### Neuron recovery, brain slice immunohistochemistry and tracing and analysis of dendritic tree morphology

Following electrophysiological recordings, slices were fixed in cold 4% paraformaldehyde overnight and then washed three times for 10 min with a 1× Tris-buffered saline (TBS) solution. They were then incubated for 1 h in a blocking solution containing 3% bovine serum albumin (BSA), 50 µM glycine, 0.05% sodium azide, and 0.3% Triton X-100 in TBS at RT. Following three 10 min washes with TBS, slices were incubated with the mouse AT180 antibody (reactive with tau phosphorylated at Thr231/Ser235; 1:2000; Thermo Scientific) in a solution containing of 0.5% BSA, 0.05% sodium azide, and 0.1% Triton X-100 in TBS for 48 h at RT. After primary antibody incubation, the slices were washed three times for 10 min with TBS and incubated in a secondary solution containing Streptavidin coupled to AlexaFluor 594 (1:2000; Life Technologies), goat anti-mouse immunoglobulin G coupled to AlexaFluor 488 (1:2000; Life Technologies), 0.1% Triton X-100, and 0.05% sodium azide in TBS overnight at RT. After a final three washes with TBS, slices were cover-slipped with Vectashield mounting medium (H-1400; Vector Laboratories Inc.) and stored at 4 °C.

The dendritic morphology of patched neurons was imaged using a confocal microscope (LSM710; Zeiss) equipped with a 20× air objective (0.8 NA; Zeiss). All images were acquired using Zen 2012 software (Zeiss). Recovered neurons were manually traced using Neurolucida software (MBF Bioscience). Neuroexplorer software (MBF Bioscience) Branch Analysis was used to calculate dendritic length and the number of branch nodes.

### Determining CA1 neuronal densities

Whole brains of rTg4510 and wild-type mice were fixed in cold 4% paraformaldehyde overnight, after which they were washed three times for 10 min with PBS solution. Sections (50 µm) were then prepared using a vibratome (Leica). Brain slices were incubated for 1 h in a blocking solution containing 0.5% BSA, 0.1% Triton X-100, and 0.05% sodium azide in PBS at RT. The slices were then incubated with mouse anti-NeuN antibody (1:500; Millipore) in blocking solution for 48 h at RT, after which they were washed again three times for 10 min with PBS and incubated in a secondary solution containing goat anti-mouse IgG coupled to AlexaFluor 488 (1:500; Life Technologies) in blocking solution for 3 h at RT. After a final three washes with PBS, the slices were cover-slipped with Vectashield mounting medium (H-1400; Vector Laboratories Inc.) and stored at 4 °C. To calculate CA1 neuronal densities the brain slices were imaged using a spinning-disk confocal system (Marianas; 3I, Inc.) consisting of an Axio Observer Z1 (Zeiss) equipped with a CSU-W1 spinning-disk head (Yokogawa Corporation of America) and an ORCA-Flash4.0 v2 sCMOS camera (Hamamatsu Photonics) with a 40× oil-immersion objective (1.4 NA; Zeiss).

### Tau expression constructs

All human tau constructs were expressed the 441-amino acid (2N4R) isoform of human tau carboxy-terminally tagged with EGFP (referred to as Tau-EGFP) in the EGFP-N1 vector (Clontech) under the control of the CMV promoter. Two mutant Tau-EGFP constructs termed E14 and A14 (all 14 serine or threonine residues T111, T153, T175, T181, S199, S202, T205, T212, T217, T231, S235, S396, S404, and S422 mutated to glutamic acid (E14) or alanine (A14), respectively) were generated by subcloning the mutant Tau fragment from pRK5-EGFP-Tau E14 (0N4R Tau, Addgene plasmid # 46907) or pRK5-EGFP-Tau AP (0N4R Tau, Addgene plasmid # 46905) into the wild-type Tau-EGFP backbone, using the Gibson assembly method (NEB E5510S). Generation of the AT180, 12E8 and PHF1 mutant tau constructs has been described previously [52]. Sanger sequencing was employed to confirm all construct sequences.

### Primary hippocampal neuron culture, immunocytochemistry and axon initial segment imaging and quantification

Hippocampal neurons from embryonic day 17 (E17) C57BL/6 mouse embryos were dissected and plated onto poly-d-lysine-coated coverslips in a 12-well plate at a density of 70,000 cells/well [9]. The plating medium was based on Neurobasal medium (Invitrogen), supplemented with 5% fetal bovine serum (Hyclone), 2% B27 (Gibco), 2 mM Glutamax (Invitrogen) and 50 U/ml penicillin/streptomycin (Invitrogen). This was changed to serum-free Neurobasal medium 24 h post-seeding, after which half of the medium was changed every three days. Neurons were transfected at 14 days in vitro (DIV 14) using Lipofectamine 2000 (Invitrogen) for either 24 or 48 h. With respect to microtubule stabilizing/destabilizing experiments, taxol (0.1 µM, Sigma) and nocodazole (0.05 µM, Sigma) were added when changing the transfection medium.

Following transfections, cells were fixed for 10 min with 4% paraformaldehyde for ankyrin G staining or 1% paraformaldehyde for Na_V_1.6 and βIV spectrin co-staining with ankyrin G. Following three 15-min washes with PBS, the cells were permeabilized in 0.25% Triton X-100 for 10 min at RT, blocked in 0.5% BSA in PBS for 1 h, and incubated overnight at RT with 0.5% BSA in PBS with one of three primary antibody configurations: (1) rabbit anti-ankyrin G (1:500, Santa Cruz) and mouse anti-Na_V_1.6 (1:200, NeuroMab); (2) rabbit anti-ankyrin G (1:500) and goat anti-βIV spectrin (1:200, Santa Cruz), or (3) rabbit anti-ankyrin G (1:500).

The co-staining of ßIV spectrin or Na_V_1.6 with ankyrin G was performed to confirm AIS localization. The cells were then washed three times for 15 min and incubated in a secondary antibody solution composed of AlexaFluor-594 (1:500, Invitrogen) for single antibody staining or AlexaFluor-594 and AlexaFluor-647 (1:500, Invitrogen) for dual staining and 0.5% BSA in PBS for 1 h at RT. Following a final three 15-min washes in PBS, cells were mounted using Vectashield mounting medium (H-1000; Vector Laboratories Inc.) and stored at 4 °C.

The AIS of stained hippocampal primary culture neurons was imaged using confocal microscopy (LSM710; Zeiss), using a 63× oil-immersion lens (1.4 NA; Zeiss), 2× digital zoom and Nyquist sampling so that each image was acquired with 16 bits per pixel and an individual pixel resolution of 0.0375 × 0.0375 × 0.13 µm^3^. Images were deconvolved using Huygens Professional software (SVI). The quantification of the AIS location and length was performed using z-stack images flattened into a single maximal projection using NIH Image J (http://www.imagej.nih.gov/ij/) and imported into MATLAB (Mathworks) for analysis using previously published custom-written functions (Matthew Grubb and Thomas Watkins, King’s College London, London, UK [8, 16]; obtained from www.mathworks.com/matlabcentral/fileexchange/28181-ais-quantification). The start and end points were calculated as the point along the axon where the normalized fluorescence intensity increased above one-third of maximal, and the mid-point was calculated as the halfway point between the identified start and end. The integrated fluorescence intensity was calculated as the area underneath the profile between the identified start and end positions using a custom MATLAB script. Axonal A14 and E14 expression levels were analyzed by converting 16-bit images to an 8-bit image and determining the mean gray value intensity of the axon, with the background mean gray value subtracted from the axonal intensity.

### AIS localization from whole-cell patch-clamp recorded neurons

Following electrophysiological recordings, slices were fixed in cold 4% paraformaldehyde overnight, washed three times for 10 min with a 1x PBS solution and incubated for 1 h in a blocking solution containing 3% bovine serum albumin (BSA), 50 µM glycine, 0.05% sodium azide, and 0.3% Triton X-100 in PBS at RT. Following three 10-min washes with PBS, the slices were incubated with Streptavidin coupled to AlexaFluor 594 (1:2000; Life Technologies) with 0.1% Triton X-100, and 0.05% sodium azide in PBS for 48 h at RT. After a further three 10-min washes with PBS, the slices were cover-slipped with Vectashield mounting medium (H-1400; Vector Laboratories Inc.) and imaged using spinning-disk confocal microscopy (20x air objective, NA 1.4) to identify the recorded neurons. Once identified, the coverslips were removed and the slices were sub-sectioned to a thickness of 80 µm and, following three 10-min washes with PBS, incubated with a rabbit anti-ankyrin G (1:500) antibody in a solution containing of 0.5% BSA, 0.05% sodium azide, and 0.1% Triton X-100 in PBS for 48 h at RT. Following the primary antibody incubation, the slices were washed three times for 10 min with PBS and incubated in a secondary solution containing Streptavidin coupled to AlexaFluor 594 (1:2000; Life Technologies) and donkey anti-rabbit immunoglobulin G coupled to AlexaFluor 488 (1:2000; Life Technologies), 0.1% Triton X-100, and 0.05% sodium azide in PBS overnight at RT. After a final three washes with PBS, slices were cover-slipped with Vectashield mounting medium (H-1400; Vector Laboratories Inc.) and stored at 4 °C. Neurons were then imaged using spinning-disk confocal (63× oil-immersion objective NA 1.4) with Nyquist sampling that enabled a voxel resolution of 0.0992 × 0.0992 × 0.130 µm^3^ (XYZ). Images were deconvolved using Huygen’s Profession software. The start of the AIS of the recorded neurons was determined using z-stack images flattened into a single maximal projection image using NIH Image J and imported into MATLAB for analysis using a previously published custom-written function, as was previously performed to determine the AIS location in primary cultured neurons.

### Statistical analysis

Statistical analysis was performed using GraphPad Prism software (v6; GraphPad Software Inc.). Statistical comparisons between groups were made using an unpaired two-tailed Student’s *t* test. A one-way ANOVA with a Sidak’s post hoc test was used for multiple comparisons. A linear regression analysis was performed to determine the relationship between AIS location and neuronal activity with *r* ^2^ values reported. An alpha value of 0.05 was used in all cases. Data are presented as mean ± SEM and individual data points.

## Results

### Depolarized action potential initiation and reduced firing in CA1 neurons of two P301L tau transgenic mouse models

To investigate if AP firing was impaired in the hippocampal CA1 region of FTLD-Tau mutant P301L tau transgenic mice, we first performed whole-cell patch-clamp recordings from rTg4510 and wild-type mice aged 1–2 months (an early stage of tauopathy prior to overt tau hyperphosphorylation and synaptic impairment), 4–6 months (a mid-stage with extensive tau hyperphosphorylation and impairment of synaptic activity and spatial memory) and 12–14 months (a late stage after synaptic loss and neurodegeneration have been initiated) [21, 41, 45]. At all ages investigated, AP firing was reduced in rTg4510 excitatory pyramidal neurons compared to the wild-type controls (Fig. 1a, b). Consistent with this observation, the threshold for AP firing was shifted in a depolarized direction and the AP amplitude was reduced in all rTg4510 neurons (Fig. 1c–e), indicating that the reduction in CA1 AP firing was due to a depolarized shift in the threshold for AP initiation. In addition to these changes, the rheobase current required to fire the initial AP was increased in rTg4510 neurons at 1–2 and 4–6 months of age, but not at 12–14 months (Fig. 1f), which may result from an increase in input resistance that was only observed at this time point (Supplementary Figure 1a–c). The AP rise-time and half-width were only reduced at 1–2 months of age and not in the older age groups, and the AHP and holding current of rTg4510 neurons were only enhanced from 4 to 6 months onwards compared to that of wild-type neurons (Supplementary Figure 2a–d), suggesting that these changes were not the cause of the reduced AP firing. Unlike what has been reported in layer 3 cortical neurons of rTg4510 mice [3], the sag potential was not altered at any age in CA1 pyramidal neurons (Supplementary Figure 2e). Taken together, these data demonstrate that the AP firing of CA1 neurons is consistently impaired in the rTg4510 mouse model due to a depolarizing shift in AP initiation.

Although the P301L tau mutation in the rTg4510 mouse model is driven by the CaMKII promoter that targets expression to excitatory neurons, we also performed patch-clamp recordings from fast-spiking CA1 inhibitory interneurons to determine if their excitability was altered. However, other than a reduction in AP rise-time at 12–14 months of age, no change in rTg4510 AP firing, AP morphology or passive properties was observed compared to wild-type controls at any age investigated (Supplementary Figure 1d–f; Supplementary Figure 3), indicating that excitatory but not inhibitory neuronal function is impaired in rTg4510 mice.

Whereas the rTg4510 mouse model faithfully recapitulates many aspects of human tauopathies, one potential limitation is the high expression level of P301L human tau, which is 13-fold higher than that of endogenous tau [45]. We therefore investigated if CA1 pyramidal neuron AP firing was also impaired in the pR5 mouse model, which harbors the same mutation but expresses mutant human tau at much lower levels, 0.7 times those of endogenous murine tau [14]. Reflecting these lower levels, tau pathology in the pR5 mice develops later and is less pronounced than that in the rTg4510 strain. For example, whereas NFT formation in pR5 mice is initiated in the amygdala at 6 months and eventually spreads to the hippocampus, in rTg4510 mice these lesions are already detectable throughout the brain at 2.5 months of age [6, 14, 41, 45]. Despite this difference in pathology, patch-clamp recordings from CA1 pyramidal neurons in brain slices from 15- to 17-month-old pR5 mice revealed a similar, although less severe AP impairment to that observed in the rTg4510 model. Although AP firing was not significantly reduced in pR5 neurons (Fig. 1a, b), the AP threshold was shifted in a depolarized direction and the AP amplitude was reduced compared to wild-type littermates, consistent with the above recordings from rTg4510 mice (Fig. 1c–e). No changes in the rheobase, AP rise-time, AP half-width, sag potential, holding current, neuronal capacitance or input resistance were observed, whereas the AHP amplitude was increased in pR5 neurons compared to wild-type controls (Fig. 1f; Supplementary Figure 1g; Supplementary Figure 2a–e). This demonstrates that, as for rTg4510 CA1 neurons, the threshold for AP initiation is shifted in a depolarized direction in the pR5 strain.

### Reduced firing in rTg4510 mice occurs in the presence of hyperphosphorylated tau prior to neurodegeneration

**Fig. 2:**
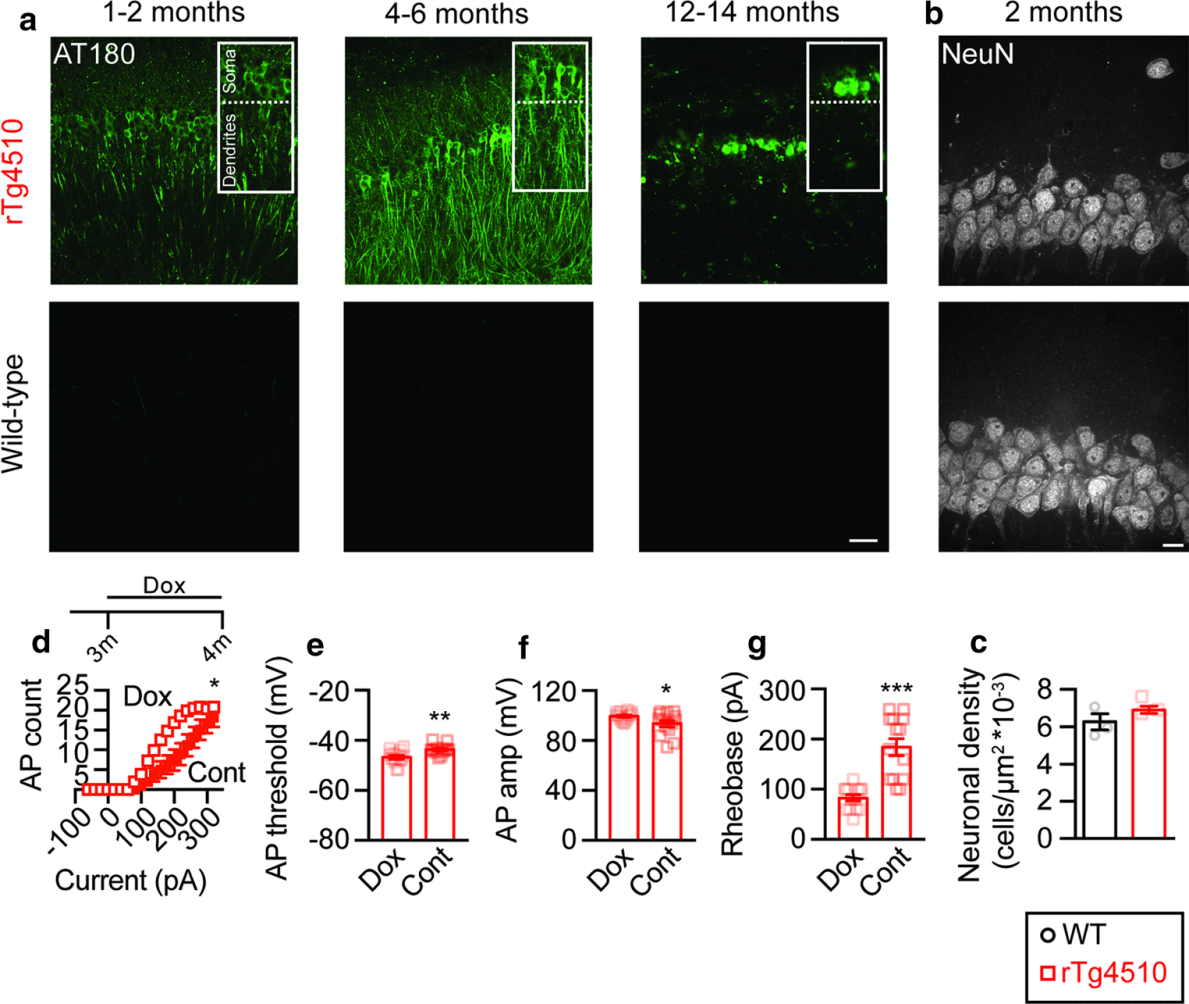
Early hyperphosphorylated tau visualized in brain slices from rTg4510 mice. **a** Representative photomicrographs demonstrating AT180 (pThr231/pSer235) staining in brain slices from rTg4510 (*top row*) and wild-type (*bottom row*) mice at 1–2 (*left*), 4–6 (*middle*) and 12–14 months of age (*right*). *Scale bar* 50 µm. **b** Representative photomicrographs of NeuN staining of rTg4510 (*top row*) and wild-type (*bottom row*) CA1 neurons in 2-month-old mice. *Scale bar* 10 µm. **c** Quantification of neuronal density in the CA1 region (*p* = 0.25, *n* = 3 mice per group). **d** Input–output relationship (rTg4510 doxycycline *n* = 14 neurons *n* = 3 mice, rTg4510 control *n* = 14 neurons *n* = 3 mice, *p* = 0.0102). **e** AP threshold (*p* = 0.0025), **f** AP amplitude (*p* = 0.0329) and **g** rheobase current (*p* < 0.0001) for rTg4510 mice fed a doxycycline-containing chow diet or control diet for 4 weeks: rTg4510 mice where 3 months of age at the beginning of the treatment. *p < 0.05, **p < 0.01, ***p < 0.001. Data presented as mean ± SEM and individual data points. Statistical comparisons were made using an unpaired two-tailed Student’s *t* test

**Fig. 3:**
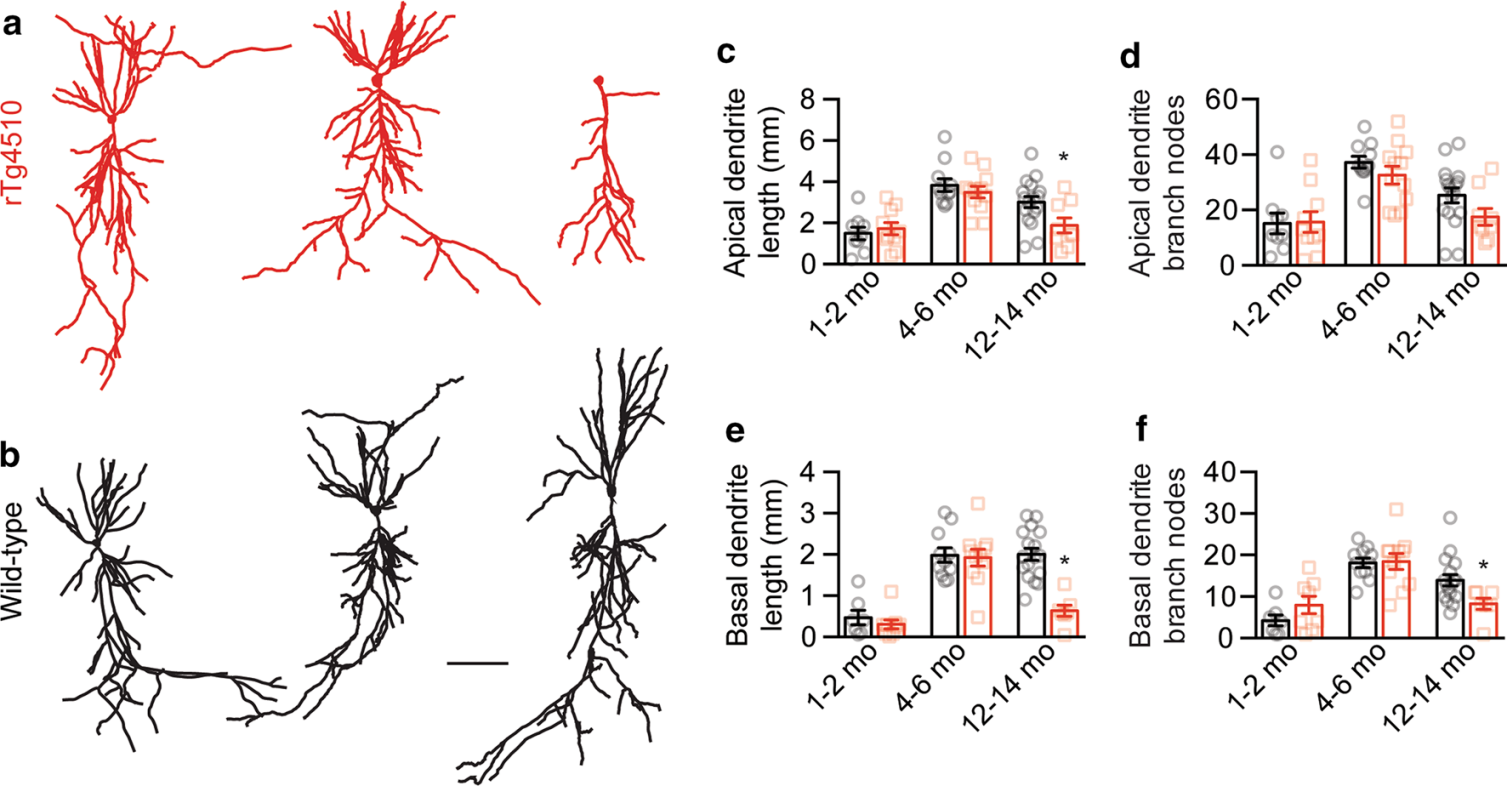
CA1 pyramidal neuron neurodegeneration occurs late in rTg4510 mice. Representative 2D projections of 3D reconstructions of **a** rTg4510 and **b** wild-type neurons at 1–2 (*left*), 4–6 (*middle*) and 12–14 months of age (*right*). **c** Total length (1–2 months: rTg4510 *n* = 10, wild-type *n* = 9, *p* = 0.58; 4–6 months: rTg4510 *n* = 12, wild-type *n* = 11, *p* = 0.42; 12–14 months: rTg4510 *n* = 9, wild-type *n* = 17, *p* = 0.0228) and **d** number of branch nodes (1–2 months: *p* = 0.93, 4–6 months: *p* = 0.24, 12–14 months: *p* = 0.08) within the CA1 pyramidal neuron apical dendritic tree, and **e** total length (1–2 months: *p* = 0.42, 4–6 months: *p* = 0.82, 12–14 months: *p* = 0.0001) and **f** number of branch nodes (1–2 months: *p* = 0.17, 4–6 months: *p* = 0.90, 12–14 months: *p* = 0.0212) from the basal dendritic trees of wild-type and rTg4510 neurons. *p < 0.05. Data presented as mean ± SEM and individual data points. *Scale bar* 100 µm. Statistical comparisons were made using an unpaired two-tailed Student’s *t* test

rTg4510 mice are characterized by the neuronal accumulation of hyperphosphorylated tau that eventually leads to neurodegeneration, which is clearly apparent by 5.5 months of age [1], whereas neuronal loss has not been reported in the pR5 model [14]. We therefore used the rTg4510 mouse model to investigate if neurodegeneration was causing the observed reduction in AP firing. We first determined the tau pathology in brain slices from rTg4510 and wild-type mice at the ages at which we had recorded. Strong immunoreactivity for hyperphosphorylated tau, as shown for the early pathological phospho-epitope AT180 (pThr231/pSer235), was observed in the CA1 pyramidal neuron layer of rTg4510 mice at all ages examined, but was absent in wild-type controls (Fig. 2a). The presence of tau hyperphosphorylation in the CA1 region of pR5 mice has previously been demonstrated [6, 50]. Furthermore, immunoreactivity for hyperphosphorylated tau was observed prior to changes in CA1 neuronal density (Fig. 2b, c), indicating that tau hyperphosphorylation and neuronal dysfunction occur before cell loss. To determine if this presence of hyperphosphorylated tau was causative in the reduction of neuronal excitability observed in the rTg4510 mouse model, we treated rTg4510 mice with doxycycline to suppress expression of the tau transgene [45]. Patch-clamp recordings revealed that feeding rTg4510 mice doxycycline for a period of four weeks was sufficient to completely reverse the impairments observed in control rTg4510 mice (Fig. 2d–g). We next assessed the dendritic morphology of the CA1 pyramidal neurons from control rTg4510 and wild-type mice from which we had recorded. We observed no change in the length or number of branch nodes in either the apical or basal tree of 1- to 2- and 4- to 6-month-old rTg4510 neurons compared to wild type (Fig. 3a–f). However, by 12–14 months of age an extensive reduction in dendritic length and complexity was observed (Fig. 3a–f). Together, these data indicate that the reduction in AP firing of CA1 pyramidal neurons is caused by the presence of hyperphosphorylated tau and is not a consequence of neurodegeneration.

### Pseudo-hyperphosphorylated tau relocates the AIS down the axon

AP initiation occurs at the AIS [39], facilitated by the high concentration of voltage-gated sodium (Na_V_) channels [10, 28, 44]. Na_V_ channel localization to the AIS is achieved through the interaction of ion channels with the AIS-specific cytoskeletal protein ankyrin G [25, 55], a commonly used AIS marker. One mechanism by which the AIS tunes neuronal excitability is by changing its location along the axon in an activity-dependent manner, such that an increase in the distance of the AIS from the soma results in reduced neuronal excitability [16]. Considering that, in tau-overexpression systems, fluorescence intensity profiles have revealed that the highest levels of tau are in the proximal axon where the AIS is located [51], we asked whether the presence of hyperphosphorylated tau could change the location of the AIS relative to the soma, which would then affect the firing properties of the neuron.

**Fig. 4:**
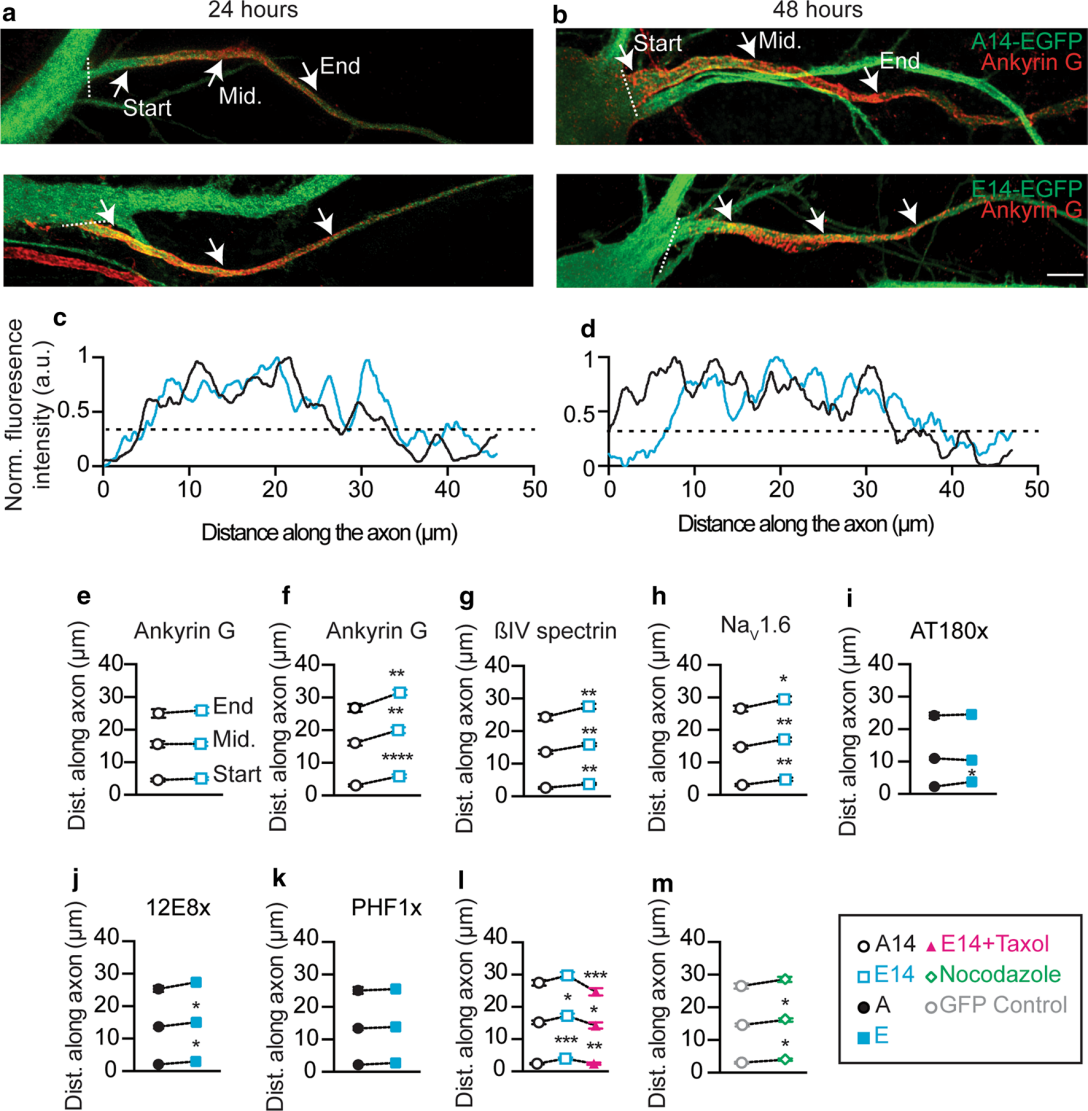
Pseudo-phosphorylated tau relocates the AIS down the axon in a process mediated by microtubules. Representative photomicrograph of A14- (*top*) and E14-tau-EGFP (*lower*) transfected hippocampal neurons stained for ankyrin G **a** 24 h and **b** 48 h after transfection. *Arrows* indicate the start, middle and end of the AIS as determined by quantitative analysis of the **c**,** d** axonal fluorescence profile. The *dashed line* indicates the normalized detection threshold. Quantification of ankyrin G labeling in neurons transfected with E14- (*blue*) and A14-tau (*black*) for **e** 24 h (start *p* = 0.48, middle *p* = 0.94, end *p* = 0.54; E14 *n* = 61, A14 *n* = 46) and **f** 48 h (start *p* = 0.0001, middle *p* = 0.0027, end *p* = 0.0017; E14 *n* = 47, A14 *n* = 62). AIS location of E14- and A14-tau-transfected neurons labeled for **g** βIV spectrin (start *p* = 0.0041, middle *p* = 0.0024, end *p* = 0.0084; E14 *n* = 55, A14 *n* = 52) and **h** Na_V_1.6 (start *p* = 0.0042, middle *p* = 0.0045, end *p* = 0.0316; E14 *n* = 53, A14 *n* = 53). Using ankyrin G staining, the role of site-specific phosphorylation in AIS relocation was determined: **i** AT180 (*start p* = 0.0138, middle *p* = 0.44, end *p* = 0.76; AT180E *n* = 53, AT180A *n* = 52), **j** 12E8 (start *p* = 0.0183, middle *p* = 0.0352, end *p* = 0.09; 12E8E *n* = 54, 12E8A *n* = 51), and **k** PFH1 (start *p* = 0.34, middle *p* = 0.56, end *p* = 0.75; PHF1E *n* = 50, PHF1A *n* = 38). **l** Treatment with 0.1 µM taxol (*magenta*) prevents AIS relocation (start, ANOVA, *F* = 8.05, *p* = 0.0005; middle, ANOVA, *F* = 4.057, *p* = 0.0191; end, ANOVA, *F* = 6.614, *p* = 0.0017; E14 *n* = 57, A14 *n* = 54, E14+ taxol *n* = 51). **m** Microtubule destabilization with 0.05 µM nocodazole (*green*) relocates the AIS (*gray*, control, start: *p* = 0.0169, middle: *p* = 0.0297, end: *p* = 0.0856; nocodazole *n* = 54, control n = 55). *p < 0.05, **p < 0.01, ***p < 0.001 and ****p ≤ 0.0001. Data presented as mean ± SEM. *Scale bar* 5 µm. Statistical comparisons were made using an unpaired two-tailed Student’s *t* test or a one-way ANOVA with a Sidak’s post hoc test

To address this, we transfected primary hippocampal neurons with either an E14 tau expression construct (with 14 critical Ser/Thr residues being replaced by glutamic acid) to mimic tau hyperphosphorylation, or a corresponding A14 control construct (where these residues were replaced by alanine) to prevent phosphorylation [21]. Both of these constructs were carboxy-terminally tagged with EGFP to facilitate the identification of transfected neurons. Interestingly, 48 h after transfection, the entire AIS of the E14-expressing neurons was relocated approximately 4–5 µm down the axon without altering the AIS length compared to A14-expressing controls, as determined by analysis of the ankyrin G fluorescence profile (Fig. 4b, d, f; Supplementary Figure 4b). In contrast, at the earlier time-point of 24 h post-transfection, no change in the location or length of the AIS was observed (Fig. 4a, c, e; Supplementary Figure 4a). As there was no difference in the expression levels of E14- and A14-tau in the axons of transfected neurons (Supplementary Data Figure 5a, b), and the location and length of the AIS were not different in A14-expressing neurons compared to GFP-transfected cells (Supplementary Figure 4c, d), we concluded that hyperphosphorylation of tau, and not simply its overexpression, had induced the relocation of the AIS. To determine if the entire AIS was relocated rather than just one of its structural elements, we next investigated the location of two additional AIS-specific proteins: the scaffolding protein ßIV spectrin and the Na_V_1.6 subunit, which is critical for AP initiation [44, 53]. Importantly, as for ankyrin G, both proteins were shifted to the same extent down the axons of E14-expressing neurons compared to A14-expressing controls (Fig. 4g, h), without altering the AIS length (Supplementary Fig. 4e, f). This shift in AIS position was not accompanied by a change in the protein level of ankyrin G, ßIV spectrin or Na_V_1.6 as determined by immunocytochemistry (Supplementary Figure 5c–e).

We next expressed phosphorylation-mimicking (E) and phosphorylation-abrogating (A) mutant forms of three tau epitopes that are known to be hyperphosphorylated in AD, AT180 (pThr231/pSer235), 12E8 (pSer262/pSer356) and PHF1 (pSer396/pSer404). Transfection with the AT180E and 12E8E mutants was sufficient to relocate the AIS down the axon, again without altering its length, using AT180A and 12E8A transfectants as controls (Fig. 4i, j; Supplementary Figure 4g), whereas the PHF1E mutant did not alter the location or length of the AIS compared to PHF1A (Fig. 4k; Supplementary Figure 4g). These results demonstrate that site-specific phosphorylation at sites such as the Thr231/Ser235 and Ser262/Ser356 epitopes are involved in the relocation of the AIS, whereas a site such as the Ser396/Ser404 epitope is not critical in this process.

One consequence of the hyperphosphorylation of tau is its detachment from microtubules and subsequent microtubule destabilization [47]. To evaluate their role in the hyperphosphorylated tau-induced relocation of the AIS, we treated E14-expressing neurons with Paclitaxel (taxol) to stabilize the microtubules. This treatment prevented the relocation of the AIS from its control position (Fig. 4l). However, treatment of wild-type EGFP-transfected neurons with nocodazole, which pharmacologically destabilizes microtubules, caused a distal shift of the AIS (Fig. 4m). The AIS length was not altered by either treatment (Supplementary Figure 4h, i). Taken together, these findings demonstrate that the hyperphosphorylated tau-mediated displacement of the AIS is microtubule-dependent.

### Pseudo-hyperphosphorylated tau reduces firing in primary neuronal cultures

**Fig. 5:**
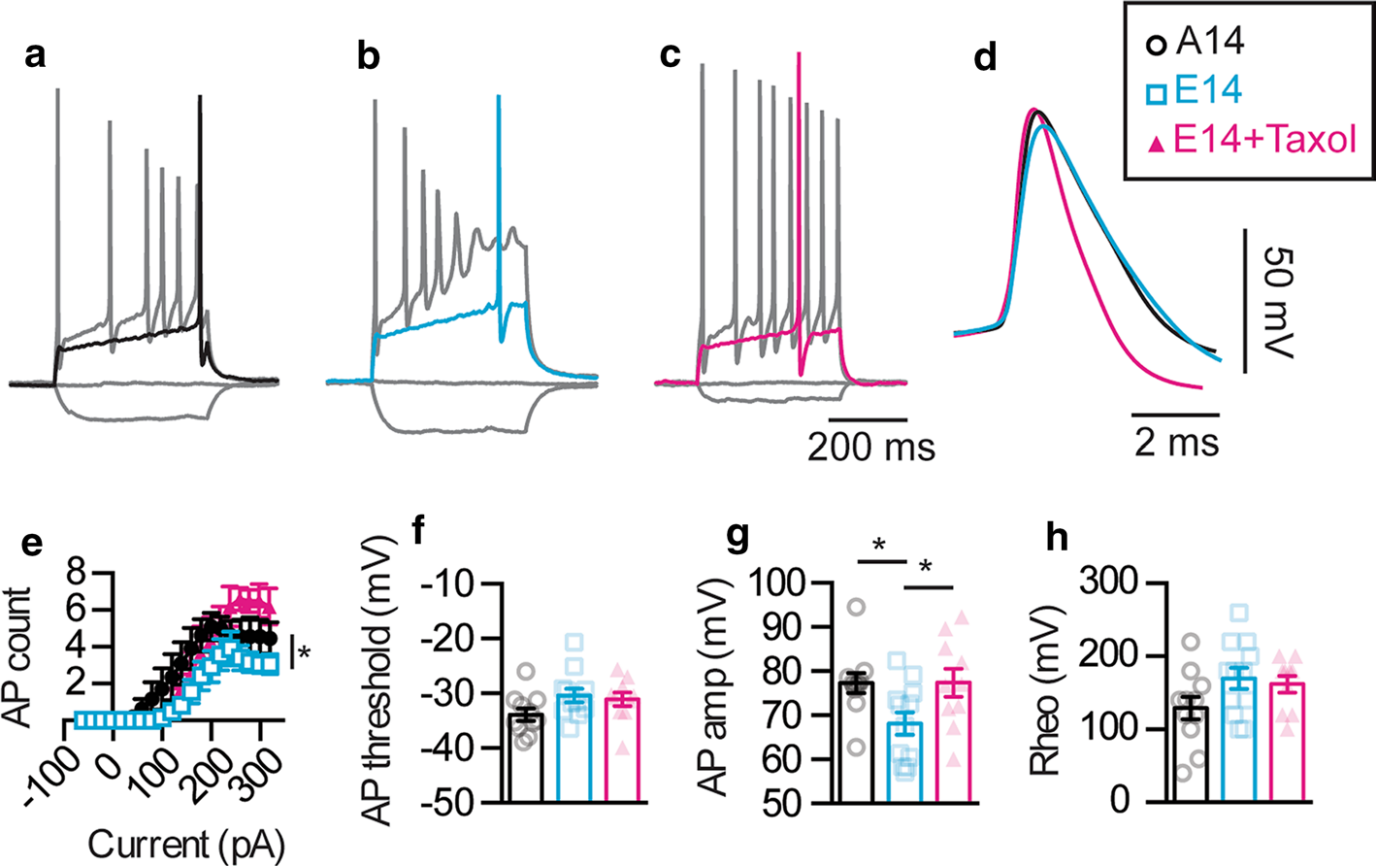
Pseudo-phosphorylated tau reduces AP firing in a similar manner to that observed in tau transgenic mouse models. Representative traces of APs fired by **a** A14- (*black*), **b** E14-transfected (*blue*) neurons and **c** E14-transfected neurons treated with 0.1 µM taxol (*magenta*) following injection of a −60, 0, rheobase and 320 pA current step. **d** Representative traces of the initial AP fired for A14, E14 and E14+ taxol neurons. Pooled data demonstrating the AP firing, including **e** input–output relationships (ANOVA, *F* = 4.084, *p* = 0.0274; E14 *n* = 12, A14 *n* = 11, E14+ taxol *n* = 10), **f** AP threshold (ANOVA, *F* = 2.439, *p* = 0.10), **g** AP amplitude (ANOVA, *F* = 4.152, *p* = 0.0256), and **h** rheobase (ANOVA, *F* = 2.4, *p* = 0.11). *p < 0.05. Data are presented as mean ± SEM and individual data points. Statistical comparisons were made using a one-way ANOVA with a Sidak’s post hoc test between all three groups

We next investigated if the observed distal shift in the position of the AIS induced by hyperphosphorylated tau alters AP firing by performing whole-cell patch-clamp recordings from E14-transfected primary neurons. This revealed a similar reduction in neuronal excitability to that previously observed in the two tau transgenic mouse models, in that both AP firing and the AP amplitude were reduced in E14- compared to A14-transfected neurons, with a trend for changes in the threshold and rheobase (Fig. 5a, b, d–h). Importantly AP firing of untransfected neurons was not altered compared to that of A14-expressing neurons but was significantly higher than that of E14-expressing neurons (Supplementary Figure 6a), demonstrating that it is the presence of hyperphosphorylated tau rather than tau overexpression that impairs neuronal excitability. The AP rise-time and half-width were increased in E14-transfected neurons, whereas no change was observed in the AHP amplitude, holding current, neuronal capacitance and input resistance (Supplementary Figure 1i; Supplementary Figure 6b–e). No changes in the firing and passive properties of E14-transfected neurons were observed at 24 h post-transfection compared to A14-transfected controls (Supplementary Figure 1h; Supplementary Figure 7). In addition, E14-transfected neurons that were treated with taxol to stabilize microtubules displayed control levels of AP firing and AP amplitude (Fig. 5; Supplementary Figure 6). Although the AP threshold, rheobase, rise-time, holding current, capacitance and input resistance were not affected by taxol treatment, the AP half-width was reduced and the AHP amplitude was increased (Fig. 5; Supplementary Figure 6). These data suggest that the distal shift in AIS location induced by hyperphosphorylated tau leads to the observed reduction in AP initiation and firing, recapitulating the phenotype observed in the two tau transgenic mouse models.

### The AIS of CA1 pyramidal neurons from rTg4510 mice are located further down the axon correlating with reduced firing

**Fig. 6:**
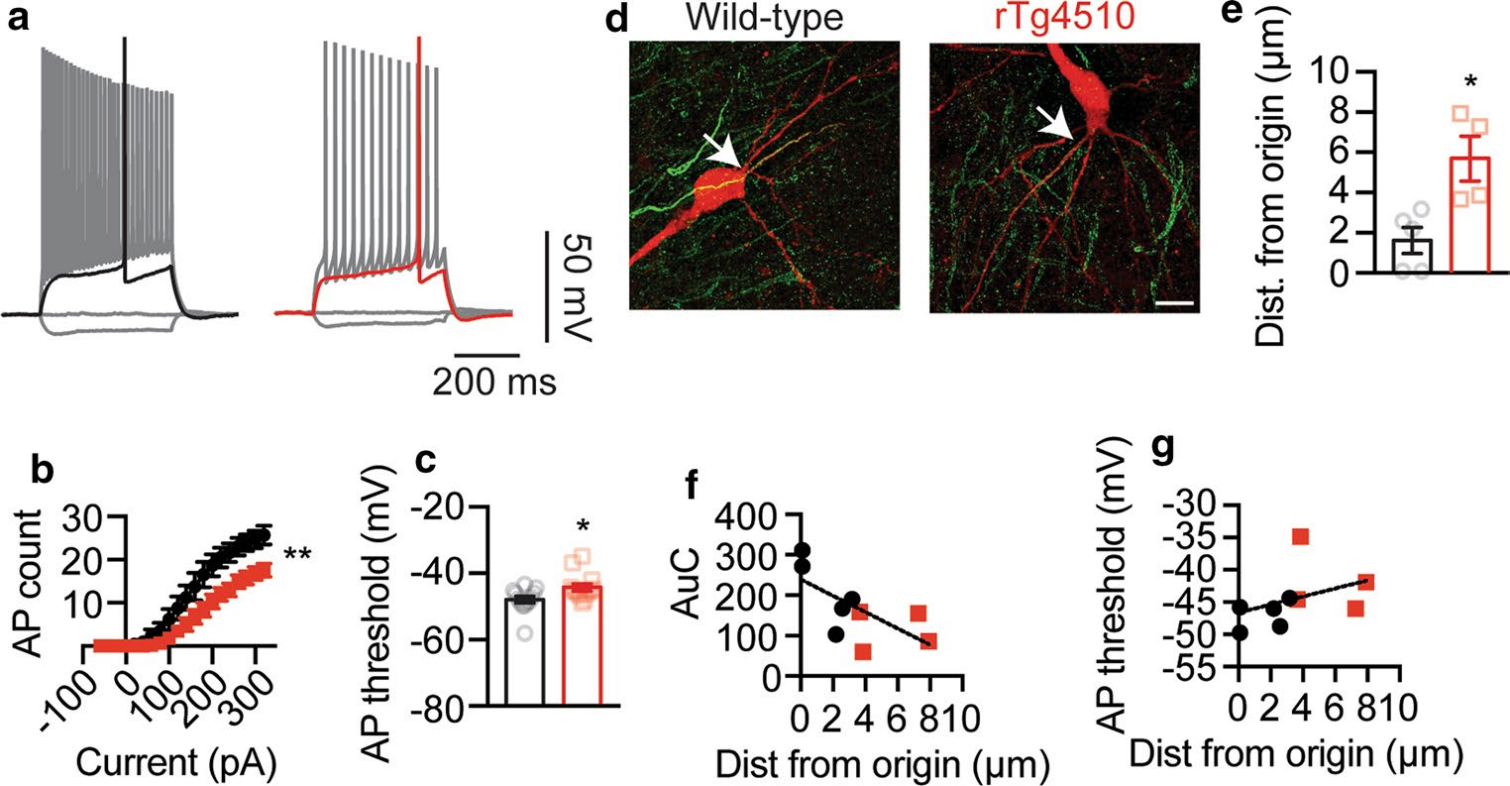
The AIS of neurons from rTg4510 mice is located further down the axon, which correlates with reduced firing and AP threshold depolarization. **a** Representative AP firing of wild-type and rTg4510 neurons following injection of a −60, 0, rheobase and 320 pA current step as well as the quantification of (**b**) input–output relationship (*p* = 0.0075, rTg4510 n = 13 neurons n = 3 mice, wild-type n = 14 neurons n = 3 mice) and **c** AP threshold (*p* = 0.0175). **d** Representative *z*-projection photomicrographs of AnkG staining (*green*) in biocytin-filled neurons (*red*) during patch-clamp recordings. *Arrows* indicate the start of the AIS. **e** Quantification of the start of the AIS, determined by fluorescence intensity, in recovered neurons (*p* = 0.0127, rTg4510 *n* = 4 neurons *n* = 3 mice, wild-type *n* = 5 neurons *n* = 3 mice). Demonstrates the relationship between the AIS location and **f** AP firing (r^2^ = 0.45) and **g** threshold for AP initiation (*r* ^2^ = 0.15). **p < 0.01 and *p < 0.05. Statistical comparisons were made using an unpaired two-tailed Student’s *t* test and a linear regression analysis

In a final series of experiments, we investigated if this distal relocation of the AIS also occurs in CA1 pyramidal neurons from 2-month-old rTg4510 mice by performing immunohistochemistry on slices in which neurons were filled biocytin during whole-cell patch-clamp recordings. Similar to the results described in Fig. 1, a reduction in AP firing and a depolarization of the threshold for AP firing were observed in rTg4510 neurons compared to wild-type (Fig. 6a–c). Furthermore, using ankyrin G labeling to identify the AIS of the filled neurons, we found that the start of the AIS of rTg4510 neurons was located further down the axon than in wild-type neurons (Fig. 6d, e). A linear relationship between both AP firing and AP threshold with the start of the AIS was observed, with the AIS location being negatively correlated with AP firing (Fig. 6f) and positively correlated with the threshold for AP initiation (Fig. 6g). Collectively, these data indicate that the distal relocation of the AIS observed in primary cultures also occurs in tau transgenic mice and contributes to the reduction in neuronal firing.

## Discussion

**Fig. 7:**
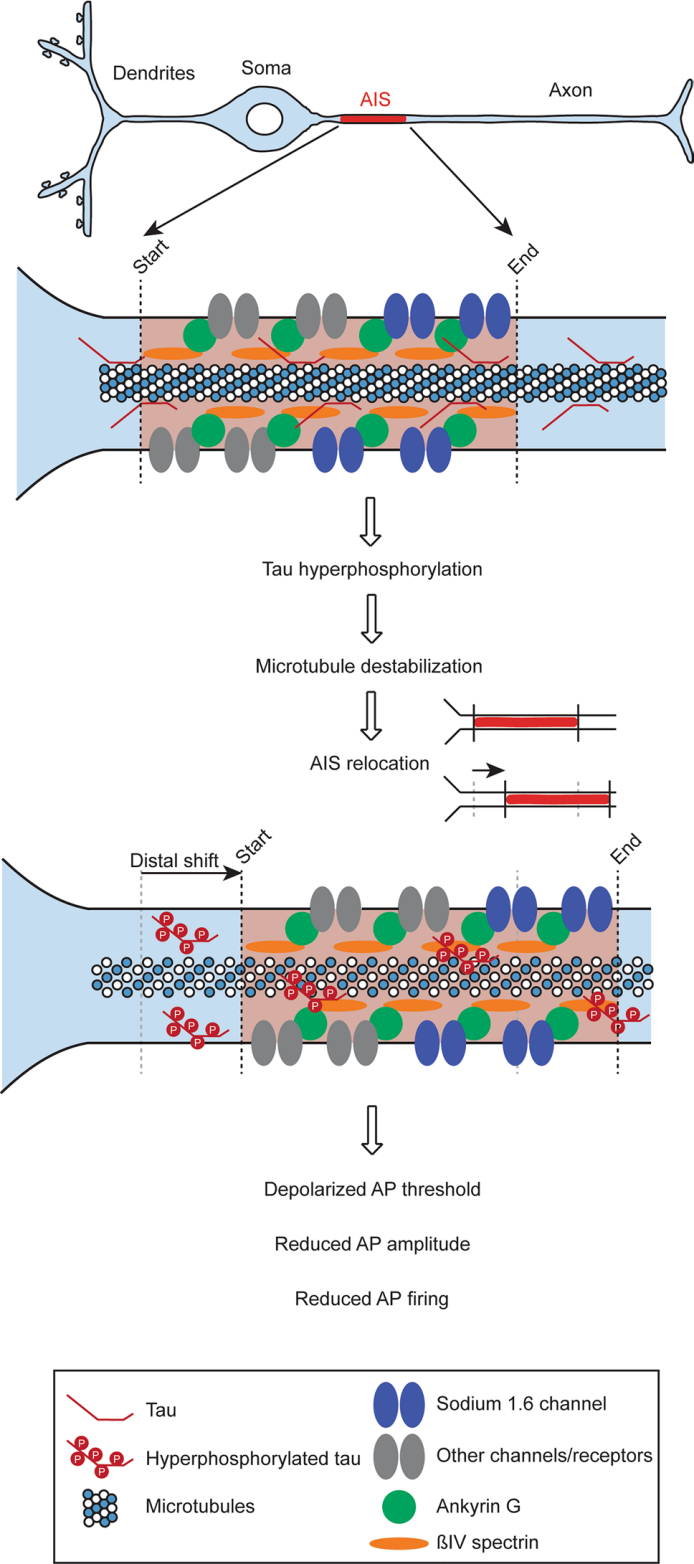
Schematic of major findings. The presence of hyperphosphorylated tau destabilizes microtubules and relocates the AIS, which in turn depolarizes the threshold for AP initiation, reducing AP amplitude and AP firing

In this study, we present a mechanism by which hyperphosphorylated tau that accumulates in tauopathies reduces neuronal excitability by destabilizing microtubules and relocating the AIS, which in turn depolarizes the threshold for AP initiation and reduces AP firing (see Fig. 7). The causal nature of this relationship is suggested by the rescue of the reduced AP firing in rTg4510 mice by suppression of pathological tau, and the finding that pharmacological stabilization of microtubules prevents both the structural and functional deficits induced by hyperphosphorylated tau, whereas pharmacological microtubule destabilization induces a relocation of the AIS.

An involvement of the AIS in neurodegenerative models has previously been demonstrated by suggesting that it can act as a retrograde barrier to axonal tau that fails in the presence of hyperphosphorylated tau and destabilized microtubules [33]. It has also been shown very recently that tau-mediated destabilization of microtubules includes the AIS [46]; however, the primary role of the AIS is in the initiation of AP firing, a function that has received little attention in neurodegeneration research. Here, we have revealed that hyperphosphorylated tau causes a reduction in AP firing by relocating the AIS, with implications for the pathogenesis of neurodegenerative diseases such as AD and FTLD-Tau. Although we cannot rule out a contribution of other changes to AP kinetics, such as AHP amplitude, to the reduction in neuronal excitability, these changes were not consistent across all models and time points investigated. Previous studies that have reported changes in the location of the AIS are predominately consistent with the homeostatic regulation of firing [16, 19, 30]. In contrast, this is not the case for the hyperphosphorylated tau-induced relocation of the AIS, which is caused by an intrinsic change in the structure of the proximal axon resulting from microtubule destabilization [46]. Interestingly, a recent study suggests that a neuron with a more proximally located AIS experiences an increased current sink into the large somatodendritic region, which reduces the rising phase of an AP thereby decreasing its activity [17]. Theoretically, the opposite should also apply, a neuron with an AIS located distally would experience a reduced current loss from the somatodendritic region and, subsequently, increased AIS activity; however, this was not experimentally observed in the current study. Why this is the case requires further investigation, although one possibility could be a change in the resistance of the small section of the axon between the AIS and the soma, due to changes in the stability of the microtubule network.

Altered excitability of cortical neurons, prior to neurodegeneration, has previously been reported in the rTg4510 mouse model, although in contrast to our findings an increase in neuronal excitability was observed [3, 43]. While this result is yet to be validated in multiple tauopathy models, it does suggest that a neuronal dysfunction resulting from tau hyperphosphorylation occurs in a brain region-specific manner. In contrast with these previous in vitro results yet similar to what we describe here, a recent publication reported that pathological tau reduces both neocortical single neuron activity and network oscillations in vivo [36].

The formation of the AIS during development is dependent on intact microtubules [26, 32, 38, 54]. Our data indicate that microtubules also play a role in determining the location of the AIS in mature neurons and may provide a direct cellular mechanism by which hyperphosphorylated tau impairs neuronal activity and possibly contributes to deficits in cognitive function. However, further investigation is required to determine how the pathological tau-mediated reduction in AP firing and previously reported impairments in synaptic activity are linked, an important step in understanding how pathological tau causes the cognitive decline observed in AD. Nonetheless, it is clear that pathological tau hyperphosphorylation alters neuronal activity well before changes in neuronal morphology and density occur.

In addition to the pathological implications, the findings described here provide novel insight into the physiological plasticity of the AIS. Relocation of the AIS has previously been reported to occur in an activity-dependent manner via the activation of L- and/or T-type calcium channels and subsequent downstream signaling that requires the calcium-activated phosphatase calcineurin [8, 16]. The data presented here indicate that relocation of the AIS requires structural changes to the microtubule network, a finding supported by a recent analysis of microtubules in the AIS [46]. Given that microtubules are modulated by pathological tau phosphorylation, it is possible that, under normal conditions, physiological tau phosphorylation plays a role in modulating the AIS, although this requires further investigation.

## Electronic supplementary material

Below is the link to the electronic supplementary material.

Supplementary Figure 1. Passive properties of recorded neurons. Pooled data demonstrating neuronal capacitance (left) and input resistance (right) for pyramidal neurons from rTg4510 mice at (**a**) 1-2 months, (**b**) 4-6 months, and (**c**) 12-14 months of age, fast-spiking inhibitory interneurons from rTg4510 mice at (**d**) 1-2 months, (**e**) 4-6 months, and (**f**) 12-14 months of age, as well as (**g**) pyramidal neurons from 15-17 month old pR5 mice and primary neurons transfected for (**h**) 24 and (**i**) 48 hours. *p<0.05. Data are presented as mean ± SEM and individual data points. Statistical comparisons were made using an unpaired two-tailed Student’s t-test or one-way ANOVA with a Sidak’s post hoc test. (TIFF 2320 kb)

Supplementary Figure 2. Changes in AP morphology in rTg4510 and pR5 mice. Pooled data demonstrating AP morphology from rTg4510 (red), pR5 (green) and wild-type (black) neurons, including (**a**) AP rise-time (top row: p=0.40; second row: p=0.22; third row: p=0.056; fourth row: p=0.46), (**b**) half-width (top row: p=0.11; second row: p=0.34; third row: p=0.20; fourth row: p=0.57), (**c**) AHP amplitude (first row: p=0.21; second row: p=0.0028,; third row: p=0.0185,: fourth row: p=0.66), (**d**) holding potential (top row: p=0.22; second row: p=0.0361; third row: p=0.0051; fourth row: p=0.40), and (**e**) sag potential (top row: p=0.75; second row: p=0.11; third row: p=0.27,; fourth row: p=0.75). *p<0.05, **p<0.01, ***p<0.001. Data are presented as mean ± SEM and individual data points. Statistical comparisons were made using an unpaired two-tailed Student’s t-test. (TIFF 2247 kb)

Supplementary Data Figure 3. Activity of CA1 inhibitory interneurons is not impaired in rTg4510 mice. Representative traces of firing from wild-type and rTg4510 fast-spiking inhibitory interneurons and input-output relationships recorded from mice at (**a, b**) 1-2 months (p=0.31; rTg4510 n=15, wild-type n=11), (**c, d**) 4-6 months (p=0.077; rTg4510 n=7, wild-type n=6) and (**e, f**) 12-14 months of age (p=0.24; rTg4510 n=13, wild-type n=16). Quantification of (**g**) AP amplitude (1-2 months: p=0.68, 4-6 months: p=0.29, 12-14 months: p=0.72), (**h**) rise-time (1-2 months: p=0.17, 4-6 months: p=0.061, 12-14 months: p=0.0157) (**i**) half-width (1-2 months: p=0.99, 4-6 months: p=0.80, 12-14 months: p=0.45), (**j**) AHP amplitude (1-2 months: p=0.34, 4-6 months: p=0.22, 12-14 months: p=0.08) and (**k**) holding current (1-2 months: p=0.96, 4-6 months: p=0.11, 12-14 months: p=0.26) from rTg4510 and wild-type neurons. *p<0.05. Data are presented as mean ± SEM and individual data points. Statistical comparisons were made using an unpaired two-tailed Student’s t-test. (TIFF 1953 kb)

Supplementary Figure 4. Transfection with pseudo-phosphorylated tau does not alter AIS length. AIS length quantified by immunofluorescence for ankyrin G at (**a**) 24 hours (p=0.12; A14 n=46, E14 n=61) and (**b**) 48 hours (p=0.32; A14 n=62, E14 n=47) post-transfection, (**c**) AIS location (start, ANOVA, F=18.12, p<0.0001; middle, ANOVA, F=13.15, p<0.0001; end, ANOVA, F=8.836, p=0.0002) and (**d**) length for A14-, E14- and untransfected neurons (ANOVA, F=0.52, p=0.60). Quantification of AIS length 48 hours post-transfection as identified by staining with (**e**) ßIV spectrin (p=0.06; A14 n=52, E14 n=55) and (**f**) Na_V_1.6 (p=0.40; A14 n=53, E14 n=53). AIS length as determined by ankyrin G labeling for transfection with (**g**) single phosphorylation site mutants (AT180: p=0.36, AT180A n=52, AT180E n=53; 12E8: p=0.32, 12E8A n=51, 12E8E n=54; PHF1: p=0.97, PHF1A n=38, PHF1E n=50), and (**h**) A14 and E14 transfections with taxol treatment (E14 versus E14+taxol: p=0.052, E14 n=57, E14+taxol n=51, A14 versus E14+taxol: p=0.16, A14 n=53, E14+taxol n=51), as well as (**i**) treatment with nocodazole in wild-type neurons (p=0.35, control n=55, nocodazole n=54). Data are presented as mean ± SEM. Statistical comparisons were made using an unpaired two-tailed Student’s t-test. (TIFF 1314 kb)

Supplementary Data Figure 5. Movement of the AIS is not associated with changes in AIS protein levels. (**a**) Representative gray scale images of A14- (top) and E14-expressing (bottom) neurons and their axons. (**b**) Quantification of mean gray value axonal fluorescence intensity following background subtraction in A14- and E14-expressing neurons (p=0.66; A14 n=57, E14 n=60). Integrated AIS fluorescence for A14- and E14-transfected neurons for (**c**) ankyrin G (p=0.42; A14 n=57, E14 n=45), (**d**) ßIV spectrum (p=0.37; A14 n=52, E14 n=55) and (**e**) Na_V_1.6 subunit immunostaining (p=0.87; A14 n=53, E14 n=53). Data are presented as mean ± SEM Scale bar: 5 µm. Statistical comparisons were made using an unpaired two-tailed Student’s t-test. (TIFF 1207 kb)

Supplementary Figure 6. Changes in AP morphology associated with transfection of pseudo-phosphorylated tau and taxol treatment. (**a**) Input-output relationship of A14- and E14-expressing and untransfected neurons (ANOVA, F=5.226, p=0.0121). Pooled data demonstrating (**b**) AP rise-time (ANOVA, F=5.367, p=0.0106), (**c**) half-width (ANOVA, F=10.22, p=0.0005), (**d**) AHP amplitude (ANOVA, F=27.7, p=<0.0001), and (**e**) holding current (ANOVA, F=0.50, p=0.61). *p<0.05, **p<0.01, ***p<0.001, and ****p≤0.0001. Data are presented as mean ± SEM and individual data points. Statistical comparisons were made using either a two-tailed unpaired Student’s t-test or a one-way ANOVA with a Sidak’s post hoc test. (TIFF 1014 kb)

Supplementary Figure 7. Neuronal excitability is not altered 24 hours after E14 transfection. Quantification of neuronal excitability demonstrating (**a**) input-output relationships (p=0.54; A14 n=10, E14 n=11), (**b**) AP threshold (p=0.07), (**c**) AP amplitude (p=0.25), (**d**) rheobase (p=0.42), (**e**) AP rise-time (p=0.73), (**f**) AP half-width (p=0.47), (**g**) AHP amplitude (p=0.09), and (**h**) holding current (p=0.06). Data are presented as mean ± SEM and individual data points. Statistical comparisons were made using an unpaired two-tailed Student’s t-test. (TIFF 1196 kb)
