# Supplementary figures and images for: Hyperphosphorylated tau causes reduced hippocampal CA1 excitability by relocating the axon initial segment
Source: Acta Neuropathol. 2017 Jan 16;133(5):717–30. doi: 10.1007/s00401-017-1674-1 (PMC5389999; doi:10.1007/s00401-017-1674-1)

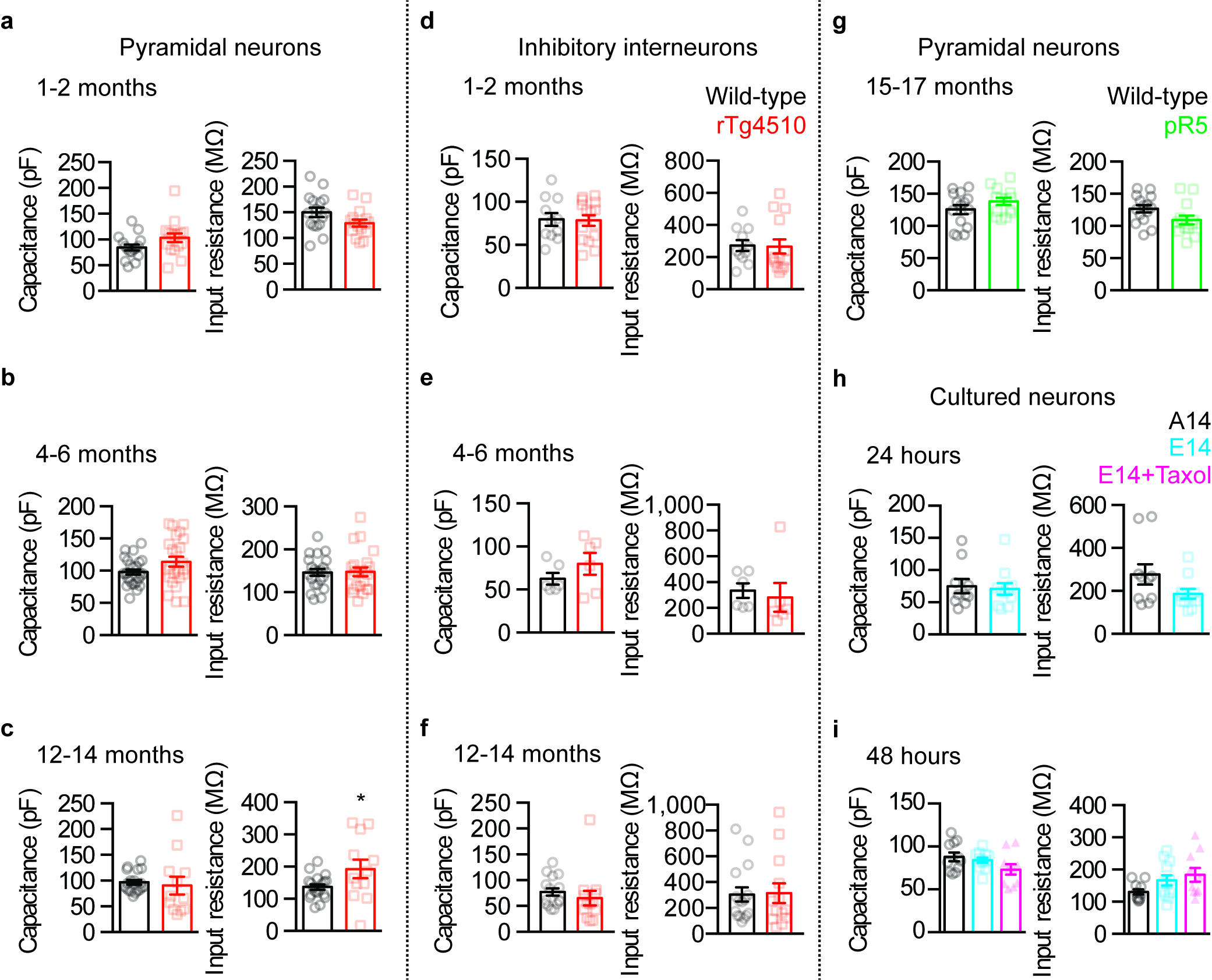

Supplement: Supplementary file 1 — Supplementary Figure 1. Passive properties of recorded neurons. Pooled data demonstrating neuronal capacitance (left) and input resistance (right) for pyramidal neurons from rTg4510 mice at (a) 1-2 months, (b) 4-6 months, and (c) 12-14 months of age, fast-spiking inhibitory interneurons from rTg4510 mice at (d) 1-2 months, (e) 4-6 months, and (f) 12-14 months of age, as well as (g) pyramidal neurons from 15-17 month old pR5 mice and primary neurons transfected for (h) 24 and (i) 48 hours. *p<0.05. Data are presented as mean ± SEM and individual data points. Statistical comparisons were made using an unpaired two-tailed Student’s t-test or one-way ANOVA with a Sidak’s post hoc test. (TIFF 2320 kb) [file 401_2017_1674_MOESM1_ESM.tif]

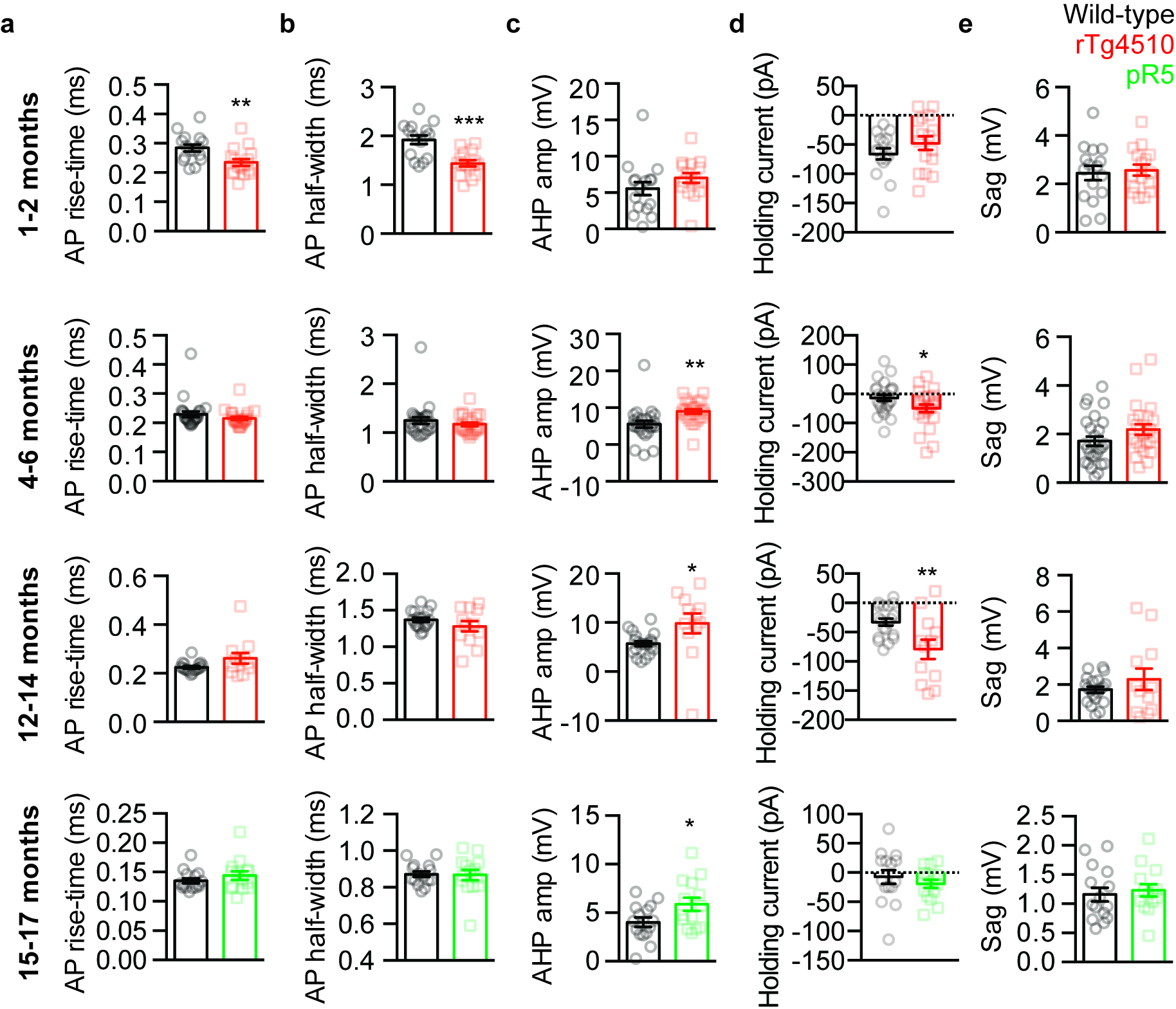

Supplement: Supplementary file 2 — Supplementary Figure 2. Changes in AP morphology in rTg4510 and pR5 mice. Pooled data demonstrating AP morphology from rTg4510 (red), pR5 (green) and wild-type (black) neurons, including (a) AP rise-time (top row: p=0.40; second row: p=0.22; third row: p=0.056; fourth row: p=0.46), (b) half-width (top row: p=0.11; second row: p=0.34; third row: p=0.20; fourth row: p=0.57), (c) AHP amplitude (first row: p=0.21; second row: p=0.0028,; third row: p=0.0185,: fourth row: p=0.66), (d) holding potential (top row: p=0.22; second row: p=0.0361; third row: p=0.0051; fourth row: p=0.40), and (e) sag potential (top row: p=0.75; second row: p=0.11; third row: p=0.27,; fourth row: p=0.75). *p<0.05, **p<0.01, ***p<0.001. Data are presented as mean ± SEM and individual data points. Statistical comparisons were made using an unpaired two-tailed Student’s t-test. (TIFF 2247 kb) [file 401_2017_1674_MOESM2_ESM.tif]

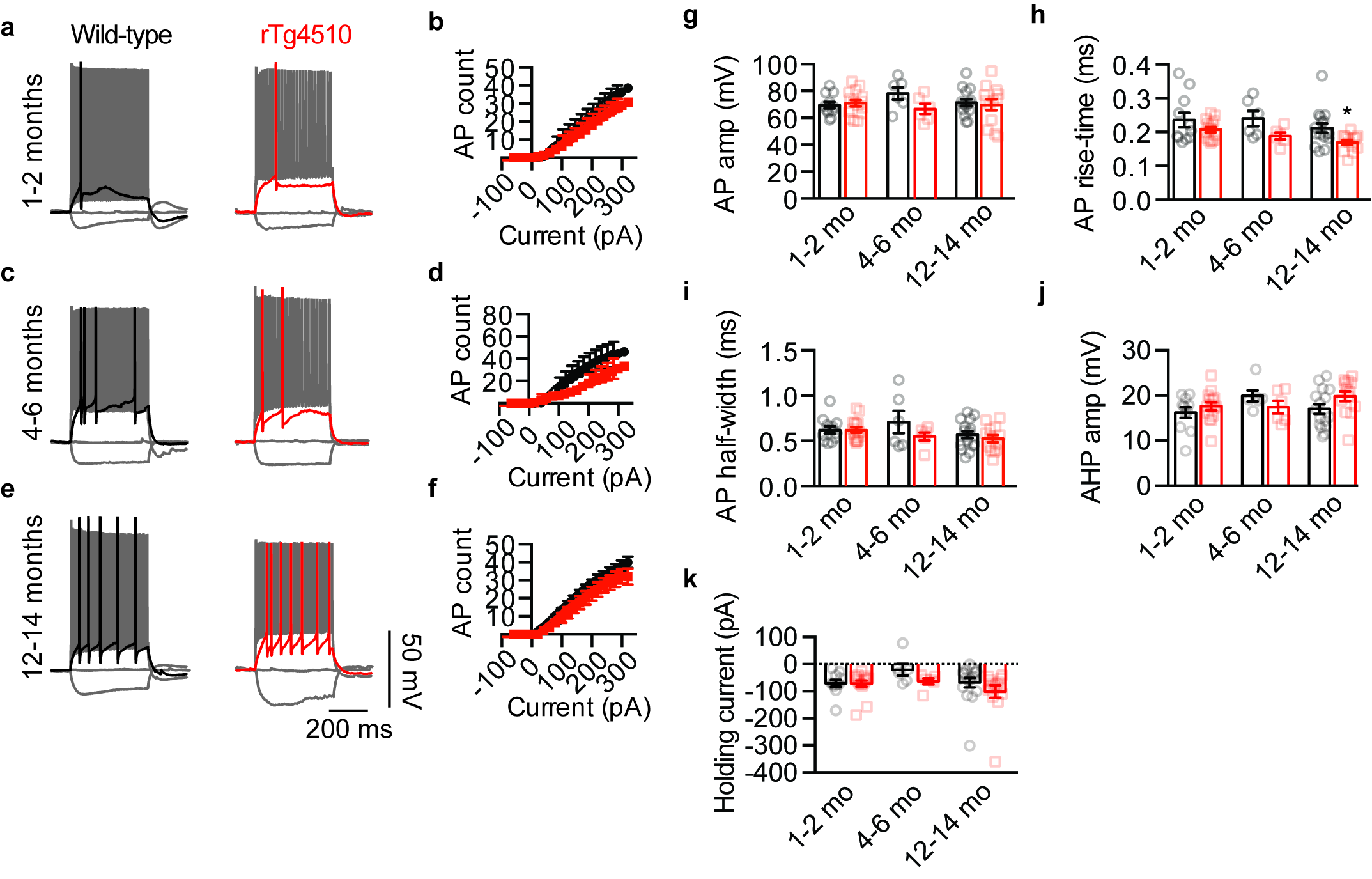

Supplement: Supplementary file 3 — Supplementary Data Figure 3. Activity of CA1 inhibitory interneurons is not impaired in rTg4510 mice. Representative traces of firing from wild-type and rTg4510 fast-spiking inhibitory interneurons and input-output relationships recorded from mice at (a, b) 1-2 months (p=0.31; rTg4510 n=15, wild-type n=11), (c, d) 4-6 months (p=0.077; rTg4510 n=7, wild-type n=6) and (e, f) 12-14 months of age (p=0.24; rTg4510 n=13, wild-type n=16). Quantification of (g) AP amplitude (1-2 months: p=0.68, 4-6 months: p=0.29, 12-14 months: p=0.72), (h) rise-time (1-2 months: p=0.17, 4-6 months: p=0.061, 12-14 months: p=0.0157) (i) half-width (1-2 months: p=0.99, 4-6 months: p=0.80, 12-14 months: p=0.45), (j) AHP amplitude (1-2 months: p=0.34, 4-6 months: p=0.22, 12-14 months: p=0.08) and (k) holding current (1-2 months: p=0.96, 4-6 months: p=0.11, 12-14 months: p=0.26) from rTg4510 and wild-type neurons. *p<0.05. Data are presented as mean ± SEM and individual data points. Statistical comparisons were made using an unpaired two-tailed Student’s t-test. (TIFF 1953 kb) [file 401_2017_1674_MOESM3_ESM.tif]

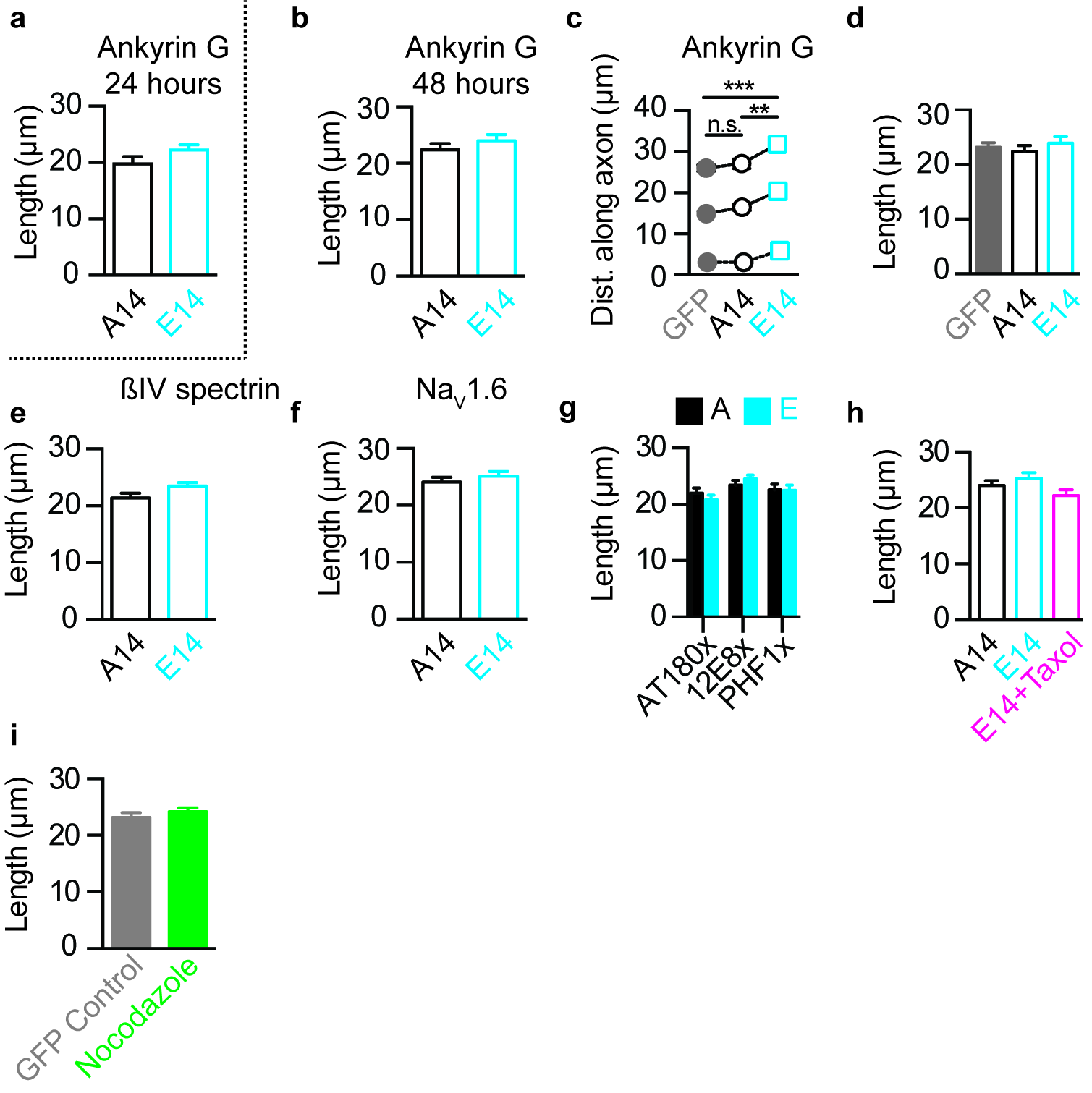

Supplement: Supplementary file 4 — Supplementary Figure 4. Transfection with pseudo-phosphorylated tau does not alter AIS length. AIS length quantified by immunofluorescence for ankyrin G at (a) 24 hours (p=0.12; A14 n=46, E14 n=61) and (b) 48 hours (p=0.32; A14 n=62, E14 n=47) post-transfection, (c) AIS location (start, ANOVA, F=18.12, p<0.0001; middle, ANOVA, F=13.15, p<0.0001; end, ANOVA, F=8.836, p=0.0002) and (d) length for A14-, E14- and untransfected neurons (ANOVA, F=0.52, p=0.60). Quantification of AIS length 48 hours post-transfection as identified by staining with (e) ßIV spectrin (p=0.06; A14 n=52, E14 n=55) and (f) NaV1.6 (p=0.40; A14 n=53, E14 n=53). AIS length as determined by ankyrin G labeling for transfection with (g) single phosphorylation site mutants (AT180: p=0.36, AT180A n=52, AT180E n=53; 12E8: p=0.32, 12E8A n=51, 12E8E n=54; PHF1: p=0.97, PHF1A n=38, PHF1E n=50), and (h) A14 and E14 transfections with taxol treatment (E14 versus E14+taxol: p=0.052, E14 n=57, E14+taxol n=51, A14 versus E14+taxol: p=0.16, A14 n=53, E14+taxol n=51), as well as (i) treatment with nocodazole in wild-type neurons (p=0.35, control n=55, nocodazole n=54). Data are presented as mean ± SEM. Statistical comparisons were made using an unpaired two-tailed Student’s t-test. (TIFF 1314 kb) [file 401_2017_1674_MOESM4_ESM.tif]

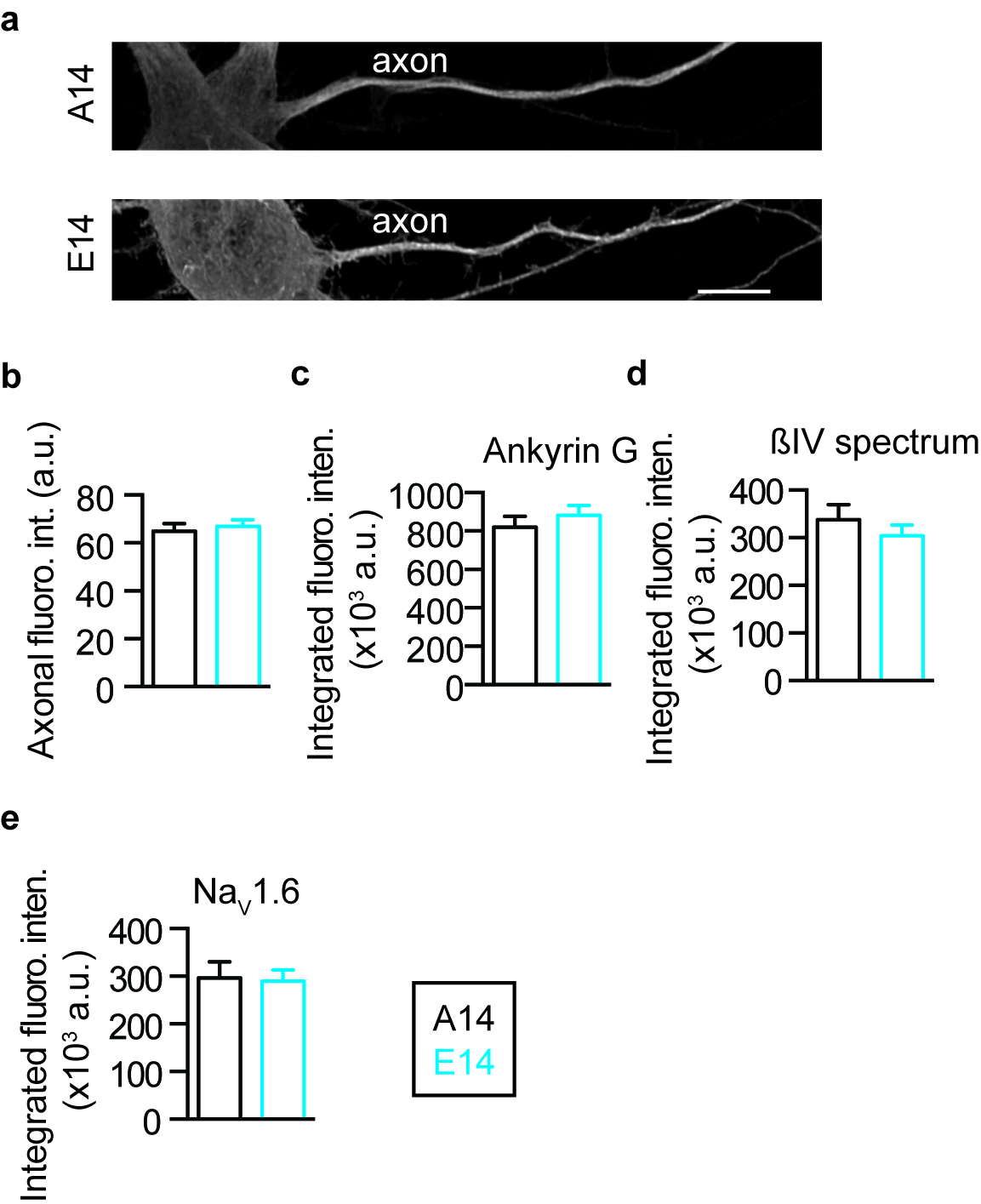

Supplement: Supplementary file 5 — Supplementary Data Figure 5. Movement of the AIS is not associated with changes in AIS protein levels. (a) Representative gray scale images of A14- (top) and E14-expressing (bottom) neurons and their axons. (b) Quantification of mean gray value axonal fluorescence intensity following background subtraction in A14- and E14-expressing neurons (p=0.66; A14 n=57, E14 n=60). Integrated AIS fluorescence for A14- and E14-transfected neurons for (c) ankyrin G (p=0.42; A14 n=57, E14 n=45), (d) ßIV spectrum (p=0.37; A14 n=52, E14 n=55) and (e) NaV1.6 subunit immunostaining (p=0.87; A14 n=53, E14 n=53). Data are presented as mean ± SEM Scale bar: 5 µm. Statistical comparisons were made using an unpaired two-tailed Student’s t-test. (TIFF 1207 kb) [file 401_2017_1674_MOESM5_ESM.tif]

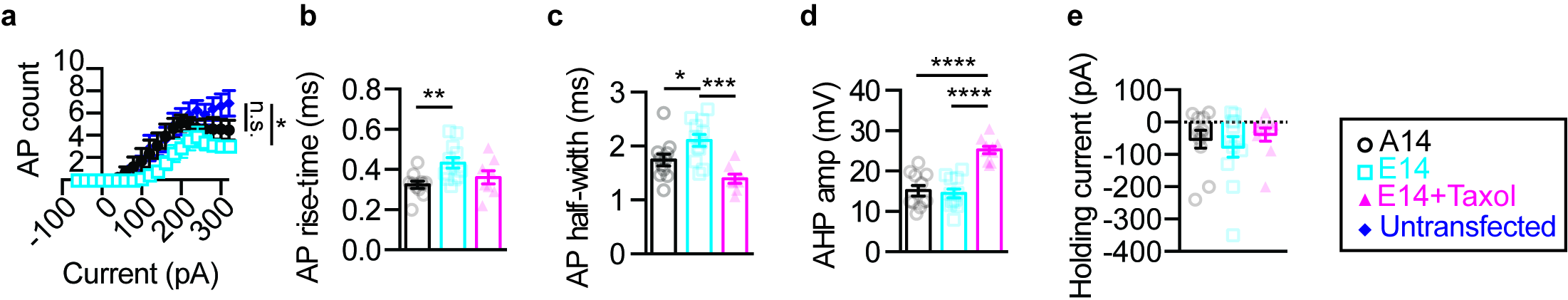

Supplement: Supplementary file 6 — Supplementary Figure 6. Changes in AP morphology associated with transfection of pseudo-phosphorylated tau and taxol treatment. (a) Input-output relationship of A14- and E14-expressing and untransfected neurons (ANOVA, F=5.226, p=0.0121). Pooled data demonstrating (b) AP rise-time (ANOVA, F=5.367, p=0.0106), (c) half-width (ANOVA, F=10.22, p=0.0005), (d) AHP amplitude (ANOVA, F=27.7, p=<0.0001), and (e) holding current (ANOVA, F=0.50, p=0.61). *p<0.05, **p<0.01, ***p<0.001, and ****p≤0.0001. Data are presented as mean ± SEM and individual data points. Statistical comparisons were made using either a two-tailed unpaired Student’s t-test or a one-way ANOVA with a Sidak’s post hoc test. (TIFF 1014 kb) [file 401_2017_1674_MOESM6_ESM.tif]

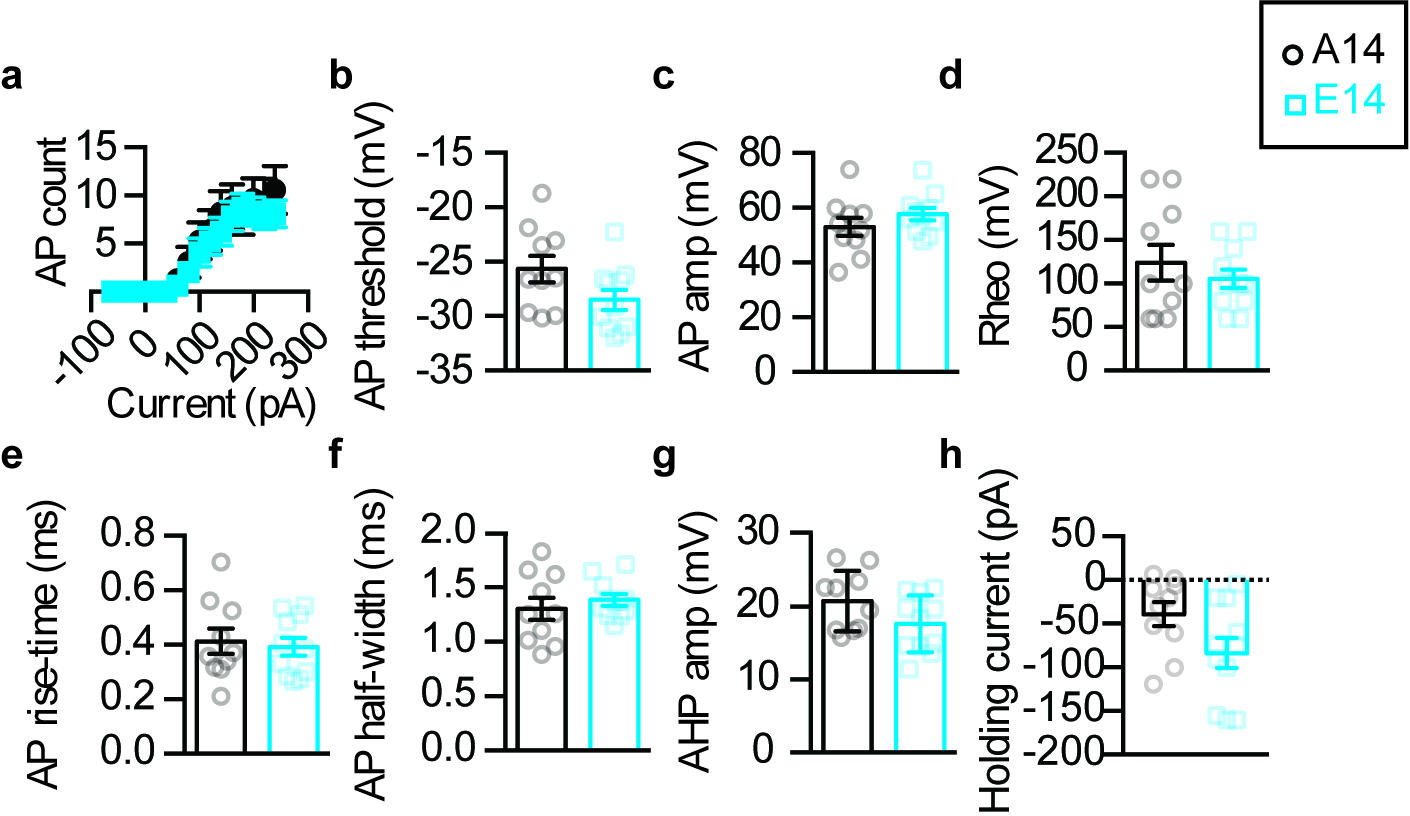

Supplement: Supplementary file 7 — Supplementary Figure 7. Neuronal excitability is not altered 24 hours after E14 transfection. Quantification of neuronal excitability demonstrating (a) input-output relationships (p=0.54; A14 n=10, E14 n=11), (b) AP threshold (p=0.07), (c) AP amplitude (p=0.25), (d) rheobase (p=0.42), (e) AP rise-time (p=0.73), (f) AP half-width (p=0.47), (g) AHP amplitude (p=0.09), and (h) holding current (p=0.06). Data are presented as mean ± SEM and individual data points. Statistical comparisons were made using an unpaired two-tailed Student’s t-test. (TIFF 1196 kb) [file 401_2017_1674_MOESM7_ESM.tif]
